# On uniform regularity and strong regularity

**DOI:** 10.1080/02331934.2018.1547383

**Published:** 2018-11-19

**Authors:** R. Cibulka, J. Preininger, T. Roubal

**Affiliations:** a Faculty of Applied Sciences, Department of Mathematics, University of West Bohemia, Pilsen, Czech Republic; b Institute of Statistics and Mathematical Methods in Economics, Vienna University of Technology, Vienna, Austria

**Keywords:** Control system, uniform metric regularity, uniform strong metric regularity, discrete approximation, path-following, 49k40, 49J40, 49J53, 90c31

## Abstract

We investigate uniform versions of (metric) regularity and strong (metric) regularity on compact subsets of Banach spaces, in particular, along continuous paths. These two properties turn out to play a key role in analyzing path-following schemes for tracking a solution trajectory of a parametric generalized equation or, more generally, of a differential generalized equation (DGE). The latter model allows us to describe in a unified way several problems in control and optimization such as differential variational inequalities and control systems with state constraints. We study two inexact path-following methods for DGEs having the order of the grid error O(h) and $O(h^{2})$, respectively. We provide numerical experiments, comparing the schemes derived, for simple problems arising in physics. Finally, we study metric regularity of mappings associated with a particular case of the DGE arising in control theory. We establish the relationship between the pointwise version of this property and its counterpart in function spaces.

## 1. Introduction

We are going to investigate uniform (metric) regularity and strong (metric) regularity on compact subsets of Banach spaces of mappings which are defined as a sum of a single-valued (possibly non-smooth) mapping and a set-valued mapping with a (locally) closed graph. In the second section, we recall basic definitions from regularity theory and derive a result guaranteeing that a perturbed problem has a solution which is similar to the classical Lyusternik-Graves and Robinson theorem. Conditions ensuring *uniform* [strong] regularity along continuous paths are obtained as a corollary. Roughly speaking, by the word ‘uniform’ we mean that the constants as well as the size of neighbourhoods, appearing in the corresponding definitions, remain the same for a certain set of mappings and/or points. These properties turn out to be the key ingredients in the proofs of the non-smooth Robinson's inverse function theorem [1] and Lyusternik-Graves theorem for the sum a Lipschitz function and a set-valued mapping with closed graph [2]. To the best of our knowledge there is no self-contained study of these properties in the literature and the results are scattered here and there.

In the third section, we study two (inexact) path-following methods for a *differential generalized equation (DGE)*, a model introduced in [3], which is a problem to find a pair of functions $x:[0,T]\to R^{n}$ and $u:[0,T]\to R^{m}$ such that

(1) $$\left\{\begin{array}{l} \dot{x}(t)=g(x(t),u(t)),\\ 0\in f(x(t),u(t))+F(u(t)),\\ x(0)=x_{{}_{I}}, \end{array}\right.\;\text{for all }t\in [0,T],$$

with a fixed *T*>0, single-valued functions $f:R^{n}\times R^{m}\to R^{d}$ and $g:R^{n}\times R^{m}\to R^{n}$, a set-valued mapping $F:R^{m}⇉R^{d}$, and a given initial state $x_{{}_{I}}\in R^{n}$. This model allows us to describe in a unified way several problems in control and optimization such as differential variational inequalities and control systems with state constraints (see [3] and references therein). The first scheme, requiring stronger smoothness properties of the solution trajectory of (1), is based on the modified Euler (Euler-Cauchy) method for solving differential equations and is shown to have the grid error of order $O(h^{2})$. On the other hand, the latter scheme, based on the Euler method, has the grid error of order O(h) but requires Lipschitz continuity of the solution trajectory only. We provide numerical experiments, comparing the schemes derived and a standard MATLAB function *ODE45*, for two simple problems arising in mechanics and electronics, respectively. The results from [3] are extended in several directions. Namely, higher-order and inexact schemes are investigated and a weaker (non-strong) metric regularity is also considered.

In the fourth section, we study regularity of mappings associated with the problem of *feasibility* in control, which is the problem to find a pair of functions $x:[0,T]\to R^{n}$ and $u:[0,T]\to R^{m}$ such that

(2) $$\dot{x}(t)=g(x(t),u(t))\;\text{and}\;f(x(t),u(t))\in U_{ad}\;\text{for a.e. }t\in [0,T],\;x(0)=0,$$

with *T*, *f* and *g* as before and a given closed convex subset $U_{ad}$ of $R^{d}$. Note that we request (2) to hold for *almost everyt* only instead of for *everyt* in (1) with $F\equiv -U_{ad}$ and $x_{I}=0$. The required ‘quality’ of the functions x(⋅) and u(⋅) will be described later in particular statements. We focus on the interplay between the pointwise conditions and their uniform and infinite-dimensional counterparts. We extend several results from [3].

*Basic notation*. The *distance* from a point *x* to a subset *A* of a metric space (X,ϱ) is $d(x,A)=\underset{y\in A}{\inf}\varrho (x,y)$. The closure and the interior of *A* is denoted by cl⁡A and int⁡A, respectively. Given sets *C*, D⊂X, the *excess* of *C* beyond *D* is defined by $e(C,D):=\underset{x\in C}{\sup}d(x,D)$. We use the convention that inf∅:=+∞ and as we work with non-negative quantities we set sup∅:=0. The closed ball centred at a point x∈X with a radius *r*>0 is denoted by ${I\;B}_{r}(x)$. A set A⊂X is *locally closed* at its point *x* if there is *r*>0 such that the set $A\cap {I\;B}_{r}(x)$ is closed. Any singleton set will be identified with its only element, that is, we write *a* instead of {a}. By F:X⇉Y we denote a set-valued mapping between sets *X* and *Y* , its *graph*, *domain*, and *range* are the sets gph⁡F:={(x,y)∈X×Y|y∈F(x)}, dom⁡F:={x∈X|F(x)≠∅}, and rge⁡F:={y∈Y|∃x∈X with y∈F(x)}, respectively. The *inverse* of *F* is a mapping $Y∋y⟼F^{-1}(y):=\{\;x\in X\;|\;y\in F(x)\}$. We write f:X→Y to emphasize that the mapping *f* is single-valued. The space of all single-valued linear continuous operators acting between Banach spaces *X* and *Y* is equipped with the standard operator norm and denoted by L(X,Y). The space $R^{n}$ is equipped with the Euclidean norm, while the Cartesian product of two or more spaces is considered with the box (product) topology. By a.e. we mean almost every in terms of the Lebesgue measure.

## 2. Uniform regularity

In our analysis, we employ two key concepts from set-valued analysis called regularity and strong regularity of a set-valued mapping. Let us emphasize that unlike definitions in [4], we prefer not to include the assumption that the mapping under consideration has a locally closed graph in any definition of regularity.

Definition 2.1 Consider metric spaces (X,ϱ), (Y,ϱ), a point $(\bar{x},\bar{y})\in X\times Y$, and a non-empty subset U×V of X×Y. A mapping F:X⇉Y is said to be

- *regular on *U* for *V** if there is a constant κ>0 such that
  $$d(x,F^{-1}(y))\leq \kappa \;d(y,F(x)\cap V)\;\text{for every }(x,y)\in U\times V;$$
- *globally regular* if *F* is regular on *X* for *Y* ;
- *regular at $\bar{x}$ for $\bar{y}$* if $\bar{y}\in F(\bar{x})$ and there are positive constants *a*, *b*, and *κ* such that
  $$d(x,F^{-1}(y))\leq \kappa \;d(y,F(x))\;\text{for each }(x,y)\in {I\;B}_{a}(\bar{x})\times {I\;B}_{b}(\bar{y}).$$
  The infimum of κ>0 such that the above inequality holds for some *a*>0 and *b*>0 is the *regularity modulus* of *F* at $\bar{x}$ for $\bar{y}$ and is denoted by $\mathrm{reg}(F;\bar{x}\;|\;\bar{y})$.

Clearly, if *F* is regular at $\bar{x}$ for $\bar{y}$ with a constant *κ* and neighbourhoods ${I\;B}_{a}(\bar{x})$ and ${I\;B}_{b}(\bar{y})$, then *F* is regular on ${I\;B}_{a}(\bar{x})$ for ${I\;B}_{b}(\bar{y})$ with the same constant. On the other hand, when the sets *U* and *V* are neighbourhoods of points $\bar{x}$ and $\bar{y}$, respectively, and $\bar{y}\in F(\bar{x})$, then regularity of *F* on *U* for *V* implies regularity of *F* at $\bar{x}$ for $\bar{y}$. The constants are the same again but neighbourhoods may differ [4, Proposition 5H.1]. By the Banach open mapping principle, a mapping A∈L(X,Y) is globally regular if and only if it is surjective.

Definition 2.2 Consider metric spaces (X,ϱ), (Y,ϱ), a point $(\bar{x},\bar{y})\in X\times Y$, and a non-empty subset U×V of X×Y. A mapping F:X⇉Y is said to be

- *strongly regular on *U* for *V** if there is a constant κ>0 such that the mapping $\sigma :V∋y⟼F^{-1}(y)\cap U$ is both single-valued and Lipschitz continuous on V=dom⁡σ with the constant *κ*;
- *strongly regular at $\bar{x}$ for $\bar{y}$* if $\bar{y}\in F(\bar{x})$ and there are neighbourhoods *U* of $\bar{x}$ and *V* of $\bar{y}$ such that *F* is strongly regular on *U* for *V*.

First, we present a statement concerning *perturbed [strong] regularity on a set*.

Theorem 2.3 Let (X,∥⋅∥) and (Y,∥⋅∥) be Banach spaces, let G:X⇉Y be a set-valued mapping, and $(\bar{x},\bar{y})\in X\times Y$. Assume that there are positive constants *a*, *b*, and *κ* such that the set $\text{gph}\;G\cap ({I\;B}_{a}(\bar{x})\times {I\;B}_{b}(\bar{y}))$ is closed in X×Y and *G* is [strongly] regular on ${I\;B}_{a}(\bar{x})$ for ${I\;B}_{b}(\bar{y})$ with the constant *κ*. Let μ>0 be such that κμ<1 and let $\kappa^{\prime }>\kappa /(1-\kappa \mu )$. Then for every positive *α* and *β* such that

(3) $$2\kappa^{\prime }\beta +\alpha \leq a\;\text{and}\;\mu (2\kappa^{\prime }\beta +\alpha )+2\beta \leq b$$

and for every mapping g:X→Y satisfying

(4) $$∥g(\bar{x})∥\leq \beta \;\text{and}\;∥g(x)-g(x^{\prime })∥\leq \mu ∥x-x^{\prime }∥\text{ for every }x,x^{\prime }\in {I\;B}_{2\kappa^{\prime }\beta +\alpha }(\bar{x}),$$

the mapping *g*+*G* has the following property: for every *y*, $y^{\prime }\in {I\;B}_{\beta }(\bar{y})$ and every $x\in (g+G{)}^{-1}(y)\cap {I\;B}_{\alpha }(\bar{x})$ there exists a [unique] point $x^{\prime }\in {I\;B}_{2\kappa^{\prime }\beta +\alpha }(\bar{x})$ such that

(5) $$y^{\prime }\in g(x^{\prime })+G(x^{\prime })\;\text{and}\;∥x-x^{\prime }∥\leq \kappa^{\prime }∥y-y^{\prime }∥.$$

Proof. We shall imitate the proof of [4, Theorem 5G.3]. First, suppose that *G* is regular on ${I\;B}_{a}(\bar{x})$ for ${I\;B}_{b}(\bar{y})$ with the constant *κ*. Choose any *α* and *β*, and then any *g* as in the statement. Then

(6) $$y-g(x)\in {I\;B}_{b}(\bar{y})\;\text{for each }(x,y)\in {I\;B}_{2\kappa^{\prime }\beta +\alpha }(\bar{x})\times {I\;B}_{\beta }(\bar{y}).$$

Indeed, fix any such a pair (x,y). Then (4) and (3) imply that

$$\begin{array}{rl} ∥y-g(x)-\bar{y}∥ & \leq ∥g(\bar{x})∥+∥g(\bar{x})-g(x)∥+∥y-\bar{y}∥\leq \beta +\mu ∥x-\bar{x}∥+\beta \\ & \leq 2\beta +\mu (2\kappa^{\prime }\beta +\alpha )\leq b. \end{array}$$

Fix any two distinct *y*, $y^{\prime }\in {I\;B}_{\beta }(\bar{y})$ and any $x\in (g+G{)}^{-1}(y)\cap {I\;B}_{\alpha }(\bar{x})$. Let $r:=\kappa^{\prime }∥y-y^{\prime }∥$. As $r\leq 2\kappa^{\prime }\beta$, the first inequality in (3) implies that

$${I\;B}_{r}(x)\subset {I\;B}_{2\kappa^{\prime }\beta +\alpha }(\bar{x})\subset {I\;B}_{a}(\bar{x}).$$

Consider the mapping

$$X∋u⟼\Phi (u)=\Phi_{y^{\prime }}(u):=G^{-1}(y^{\prime }-g(u))\subset X.$$

It suffices to show that there is a fixed point $x^{\prime }$ of Φ in ${I\;B}_{r}(x)$, because then $x^{\prime }\in (g+G{)}^{-1}(y^{\prime })$ and the desired distance estimate holds. To obtain such a point $x^{\prime }$ we are going to apply [4, Theorem 5E.2]. The set $\Omega :=\text{gph}\;\Phi \cap ({I\;B}_{r}(x)\times {I\;B}_{r}(x))$ is closed. Indeed, pick any sequence $\left(x_{n},z_{n}\right)$ in Ω converging to a point $(\tilde{x},\tilde{z})\in X\times X$. Clearly, $\left(\tilde{x},\tilde{z})\in {I\;B}_{r}(x)\times {I\;B}_{r}(x\right)$. The definition of Φ and (6) imply that

$$\begin{array}{rl} & (z_{n},y^{\prime }-g(x_{n}))\in \text{gph}\;G\cap ({I\;B}_{r}(x)\times {I\;B}_{b}(\bar{y}))\subset \text{gph}\;G\cap ({I\;B}_{a}(\bar{x})\times {I\;B}_{b}(\bar{y}))\\ & \;\text{for each }n\in N. \end{array}$$

Passing to the limit we get that $(\tilde{z},y^{\prime }-g(\tilde{x}))\in \text{gph}\;G$, that is, $(\tilde{x},\tilde{z})\in \text{gph}\;\Phi$.

According to (6) we have $y-g(x)\in G(x)\cap {I\;B}_{b}(\bar{y})$ and $y^{\prime }-g(x)\in {I\;B}_{b}(\bar{y})$, thus regularity of *G* on ${I\;B}_{a}(\bar{x})$ for ${I\;B}_{b}(\bar{y})$ yields that

$$\begin{array}{rl} d(x,\Phi (x)) & =d(x,G^{-1}(y^{\prime }-g(x)))\leq \kappa \;d(y^{\prime }-g(x),G(x)\cap {I\;B}_{b}(\bar{y}))\leq \kappa ∥y-y^{\prime }∥\\ & <\kappa^{\prime }∥y-y^{\prime }∥(1-\kappa \mu )=r(1-\kappa \mu ). \end{array}$$

Let *u*, $v\in {I\;B}_{r}(x)$ be arbitrary. Pick an arbitrary $w\in \Phi (u)\cap {I\;B}_{r}(x)$ (if there is any). As $y^{\prime }-g(u)\in G(w)\cap {I\;B}_{b}(\bar{y})$ and $y^{\prime }-g(v)\in {I\;B}_{b}(\bar{y})$, we get

$$\begin{array}{rl} d(w,\Phi (v)) & =d(w,G^{-1}(y^{\prime }-g(v)))\leq \kappa \;d(y^{\prime }-g(v),G(w)\cap {I\;B}_{b}(\bar{y}))\\ & \;\leq \kappa ∥g(u)-g(v)∥. \end{array}$$

This means that

$$\begin{array}{rl} & e(\Phi (u)\cap {I\;B}_{r}(x),\Phi (v))\leq \kappa ∥g(u)-g(v)∥\leq \kappa \mu \;∥u-v∥\\ & \;\text{whenever }u,v\in {I\;B}_{r}(x). \end{array}$$

The assumptions of [4, Theorem 5E.2] are verified. The existence of $x^{\prime }\in {I\;B}_{2\kappa^{\prime }\beta +\alpha }(\bar{x})$ satisfying (5) is established.

Now, let *G* be strongly regular on ${I\;B}_{a}(\bar{x})$ for ${I\;B}_{b}(\bar{y})$ with the constant *κ*. To prove the uniqueness, it is enough to show that the mapping ${I\;B}_{\beta }(\bar{y})∋y⟼\sigma (y):=(g+G{)}^{-1}(y)\cap {I\;B}_{2\kappa^{\prime }\beta +\alpha }(\bar{x})$ is nowhere multivalued. Assume on the contrary that for some $y\in {I\;B}_{\beta }(\bar{y})$ there are two distinct points $x_{1}$, $x_{2}\in \sigma (y)$. Clearly, $x_{1}\in G^{-1}(y-g(x_{1}))\cap {I\;B}_{a}(\bar{x})$ and $x_{2}\in G^{-1}(y-g(x_{2}))\cap {I\;B}_{a}(\bar{x})$. By (6), the points $y-g(x_{1})$ and $y-g(x_{2})$ are in ${I\;B}_{b}(\bar{y})$. Hence $0<∥x_{1}-x_{2}∥\leq \kappa ∥g(x_{1})-g(x_{2})∥\leq \kappa \mu ∥x_{1}-x_{2}∥<∥x_{1}-x_{2}∥$, a contradiction.

If, in addition to the assumptions of Theorem 2.3, we have $(\bar{x},\bar{y})\in \mathrm{gph}G$, then we arrive at [5, Theorem 2.3] which is a slight improvement [4, Theorem 5G.3], where it is supposed that *G* is regular at $\bar{x}$ for $\bar{y}$ with the constant *κ* and neighbourhoods ${I\;B}_{a}(\bar{x})$ and ${I\;B}_{b}(\bar{y})$.

Remark 2.4 Under the strong regularity, the reasoning used at the end of the proof of Theorem 2.3 implies that the function *σ*, defined therein, is Lipschitz continuous relative to $\mathrm{dom}\sigma \subset {I\;B}_{\beta }(\bar{y})$ with the constant $\kappa^{\prime }$. If, in addition,

(7) $$({I\;B}_{\alpha }(\bar{x})\times {I\;B}_{\beta }(\bar{y}))\cap \mathrm{gph}(g+G)\neq \emptyset ,$$

then $\mathrm{dom}\sigma ={I\;B}_{\beta }(\bar{y})$ and consequently *g*+*G* is strongly regular on ${I\;B}_{2\kappa^{\prime }\beta +\alpha }(\bar{x})$ for ${I\;B}_{\beta }(\bar{y})$. Note that (7) holds, for example, when $(\bar{x},\bar{y})\in \mathrm{gph}G$.

We also get the following uniformity result.

Corollary 2.5 Under assumptions of Theorem 2.3, let γ∈[0,α),δ∈[0,β), and $\left(x,y)\in {I\;B}_{\gamma }(\bar{x})\times {I\;B}_{\delta }(\bar{y}\right)$ be arbitrary. Then the mapping *g*+*G* is regular on ${I\;B}_{\alpha -\gamma }(x)$ for ${I\;B}_{\beta -\delta }(y)$ with the constant $\kappa^{\prime }$.

Proof. Let constants *γ* and *δ* along with a pair (x,y) be as in the premise. Set $U:={I\;B}_{\alpha -\gamma }(x)$ and $V:={I\;B}_{\beta -\delta }(y)$. We have to show that

$$d(u,(g+G{)}^{-1}(v))\leq \kappa^{\prime }\;d(v,(g+G)(u)\cap V)\text{ for every }(u,v)\in U\times V.$$

Fix any such a pair (u,v). Pick an arbitrary $v^{\prime }\in (g+G)(u)\cap V$ (if there is any). Noting that $U\times V\subset {I\;B}_{\alpha }(\bar{x})\times {I\;B}_{\beta }(\bar{y})$, Theorem 2.3 yields $u^{\prime }\in (g+G{)}^{-1}(v)$ with $∥u-u^{\prime }∥\leq \kappa^{\prime }∥v-v^{\prime }∥$. Hence $d(u,(g+G{)}^{-1}(v))\leq ∥u-u^{\prime }∥\leq \kappa^{\prime }∥v-v^{\prime }∥$. As $v^{\prime }\in (g+G)(u)\cap V$ was arbitrary, the proof is finished.

We show now that the regularity at each point of a compact set implies *uniform* regularity, that is, we can choose the same constant and neighbourhoods for all points in this set.

Theorem 2.6 Let (P,ϱ) be a metric space, let (X,∥⋅∥) and (Y,∥⋅∥) be Banach spaces, and let Ω be a compact subset of P×X. Consider a set-valued mapping F:X⇉Y and a mapping σ:P×X→Y such that

- for each z=(p,x)∈Ω the mapping $X∋v⟼G_{p}(v):=\sigma (p,v)+F(v)\subset Y$ has a locally closed graph at (x,0) and is [strongly] regular at *x* for 0;
- for each z=(p,x)∈Ω and each μ>0 there is δ>0 such that for each *v*, $v^{\prime }\in {I\;B}_{\delta }(x)$ and each $p^{\prime }\in {I\;B}_{\delta }(p)$ we have
  $$∥[\sigma (p^{\prime },v^{\prime })-\sigma (p,v^{\prime })]-[\sigma (p^{\prime },v)-\sigma (p,v)]∥\leq \mu ∥v-v^{\prime }∥;$$
- for each x∈X the function σ(⋅,x) is continuous.

Then there are positive constants *a*, *b*, and *κ* such that for each z=(p,x)∈Ω the mapping $G_{p}$ is [strongly] regular at *x* for 0 with the constant *κ* and neighbourhoods ${I\;B}_{a}(x)$ and ${I\;B}_{b}(0)$.

Proof. Fix any $\bar{z}=(\bar{p},\bar{x})\in \Omega$. Using (i), we find positive constants $a_{\bar{z}}$, $b_{\bar{z}}$, and $\kappa_{\bar{z}}$ such that the set $\mathrm{gph}G_{\bar{p}}\cap ({I\;B}_{a_{\bar{z}}}(\bar{x})\times {I\;B}_{b_{\bar{z}}}(0))$ is closed in X×Y and $G_{\bar{p}}$ is regular on ${I\;B}_{a_{\bar{z}}}(\bar{x})$ for ${I\;B}_{b_{\bar{z}}}(0)$ with the constant $\kappa_{\bar{z}}$. Let $\mu_{\bar{z}}:=1/(2\kappa_{\bar{z}})$ and $\kappa_{\bar{z}}^{\prime }:=3\kappa_{\bar{z}}$. Then $\kappa_{\bar{z}}\mu_{\bar{z}}<1$ and $\kappa_{\bar{z}}^{\prime }>2\kappa_{\bar{z}}=\kappa_{\bar{z}}/(1-\kappa_{\bar{z}}\mu_{\bar{z}})$. In view of (ii), there is $\alpha_{\bar{z}}\in (0,\min\{a_{\bar{z}}/2,3\kappa_{\bar{z}}b_{\bar{z}}/4\})$ such that for each *v*, $v^{\prime }\in {I\;B}_{2\alpha_{\bar{z}}}(\bar{x})$ and each $p\in {I\;B}_{\alpha_{\bar{z}}}(\bar{p})$ we have

(8) $$∥[\sigma (p,v)-\sigma (\bar{p},v)]-[\sigma (p,v^{\prime })-\sigma (\bar{p},v^{\prime })]∥\leq \mu_{\bar{z}}∥v-v^{\prime }∥.$$

Let $\beta_{\bar{z}}:=\alpha_{\bar{z}}/(2\kappa_{\bar{z}}^{\prime })$. Then

(9) $$\begin{array}{rl} 2\kappa_{\bar{z}}^{\prime }\beta_{\bar{z}}+\alpha_{\bar{z}} & =2\alpha_{\bar{z}}<a_{\bar{z}}\;\text{and}\;\mu_{\bar{z}}(2\kappa_{\bar{z}}^{\prime }\beta_{\bar{z}}+\alpha_{\bar{z}})+2\beta_{\bar{z}}\\ & =\frac{\alpha_{\bar{z}}}{\kappa_{\bar{z}}}+\frac{\alpha_{\bar{z}}}{3\kappa_{\bar{z}}}=\frac{4\alpha_{\bar{z}}}{3\kappa_{\bar{z}}}<b_{\bar{z}}. \end{array}$$

Now, (iii) implies that there is $r_{\bar{z}}\in (0,\alpha_{\bar{z}}/2)$ such that

(10) $$∥\sigma (p,\bar{x})-\sigma (\bar{p},\bar{x})∥\leq \beta_{\bar{z}}\;\text{for all}\;p\in {I\;B}_{r_{\bar{z}}}(\bar{p}).$$

Pick any $z=(p,x)\in (\mathrm{int}{I\;B}_{r_{\bar{z}}}(\bar{p})\times \mathrm{int}{I\;B}_{r_{\bar{z}}}(\bar{x}))\cap \Omega$. Define a mapping $g_{p,\bar{p}}:X\to Y$ by

$$g_{p,\bar{p}}(v):=\sigma (p,v)-\sigma (\bar{p},v),\;v\in X.$$

Then $G_{p}=G_{\bar{p}}+g_{p,\bar{p}}$. By (8), for any *v*, $v^{\prime }\in {I\;B}_{2\alpha_{\bar{z}}}(\bar{x})$ we have

$$∥g_{p,\bar{p}}(v)-g_{p,\bar{p}}(v^{\prime })∥\leq \mu_{\bar{z}}∥v-v^{\prime }∥.$$

Using (10) we get $∥g_{p,\bar{p}}(\bar{x})∥\leq \beta_{\bar{z}}$. Applying Theorem 2.3 we conclude that the following claim holds: *for every *y*, $y^{\prime }\in {I\;B}_{\beta_{\bar{z}}}(0)$ and every $v\in G_{p}^{-1}(y^{\prime })\cap {I\;B}_{\alpha_{\bar{z}}}(\bar{x})$ there exists $v^{\prime }\in G_{p}^{-1}(y)$ such that $∥v-v^{\prime }∥\leq \kappa_{\bar{z}}^{\prime }\;∥y-y^{\prime }∥$.*

As z∈Ω, we have $0\in G_{p}(x)$. We show next that

(11) $$d(v,G_{p}^{-1}(y))\leq \kappa_{\bar{z}}^{\prime }\;d(y,G_{p}(v))\;\text{for all }(v,y)\in {I\;B}_{\kappa_{\bar{z}}^{\prime }\beta_{\bar{z}}/3}(x)\times {I\;B}_{\beta_{\bar{z}}/3}(0).$$

To see this fix any such a pair (v,y). Pick an arbitrary $y^{\prime }\in G_{p}(v)$ (if there is any). The choice of $\beta_{\bar{z}}$ and $r_{\bar{z}}$ implies that

$${I\;B}_{\kappa_{\bar{z}}^{\prime }\beta_{\bar{z}}}(x)={I\;B}_{\alpha_{\bar{z}}/2}(x)\subset {I\;B}_{\alpha_{\bar{z}}}(\bar{x}).$$

First, assume that $∥y^{\prime }∥\leq \beta_{\bar{z}}$. The claim yields $v^{\prime }\in G_{p}^{-1}(y)$ with $∥v-v^{\prime }∥\leq \kappa_{\bar{z}}^{\prime }\;∥y-y^{\prime }∥$. Consequently,

$$d(v,G_{p}^{-1}(y))\leq ∥v-v^{\prime }∥\leq \kappa_{\bar{z}}^{\prime }\;∥y-y^{\prime }∥.$$

On the other hand, assuming that $∥y^{\prime }∥>\beta_{\bar{z}}$, we have $∥y^{\prime }-y∥>\beta_{\bar{z}}-\beta_{\bar{z}}/3=2\beta_{\bar{z}}/3$. Then using the claim, with $\left(y^{\prime },v):=(0,x\right)$, we find $v^{\prime }\in G_{p}^{-1}(y)$ such that $∥x-v^{\prime }∥\leq \kappa_{\bar{z}}^{\prime }\;∥y∥$. Consequently,

$$\begin{array}{rl} d(v,G_{p}^{-1}(y)) & \leq ∥v-x∥+d(x,G_{p}^{-1}(y))\leq ∥v-x∥+∥x-v^{\prime }∥\\ & \;\leq ∥v-x∥+\kappa_{\bar{z}}^{\prime }\;∥y∥\\ & \leq \kappa_{\bar{z}}^{\prime }\beta_{\bar{z}}/3+\kappa_{\bar{z}}^{\prime }\beta_{\bar{z}}/3=2\kappa_{\bar{z}}^{\prime }\beta_{\bar{z}}/3<\kappa_{\bar{z}}^{\prime }∥y-y^{\prime }∥. \end{array}$$

We have shown that $d(v,G_{p}^{-1}(y))\leq \kappa_{\bar{z}}^{\prime }\;∥y-y^{\prime }∥$ for any $y^{\prime }\in G_{p}(v)$, which proves (11).

Summarizing, for each $z=(p,x)\in (\mathrm{int}{I\;B}_{r_{\bar{z}}}(\bar{p})\times \mathrm{int}{I\;B}_{r_{\bar{z}}}(\bar{x}))\cap \Omega$ the mapping $G_{p}$ is regular at *x* for 0 with the constant $\kappa_{\bar{z}}^{\prime }$ and neighbourhoods ${I\;B}_{\kappa_{\bar{z}}^{\prime }\beta_{\bar{z}}/3}(x)$ and ${I\;B}_{\beta_{\bar{z}}/3}(0)$, that is, the size of neighbourhoods and the constant of regularity are independent of *z* in a vicinity of $\bar{z}$. From the open covering $\cup_{\bar{z}=(\bar{p},\bar{x})\in \Omega }([\mathrm{int}{I\;B}_{r_{\bar{z}}}(\bar{p})\times \mathrm{int}{I\;B}_{r_{\bar{z}}}(\bar{x})]\cap \Omega )$ of Ω choose a finite subcovering $O_{i}:=[\mathrm{int}{I\;B}_{r_{{\bar{z}}_{i}}}({\bar{p}}_{i})\times \mathrm{int}{I\;B}_{r_{{\bar{z}}_{i}}}({\bar{x}}_{i})]\cap \Omega$, i=1,2,…,k. Let $a=\min\{\kappa_{{\bar{z}}_{i}}^{\prime }\beta_{{\bar{z}}_{i}}/3|i=1,\ldots ,k\}$, $\kappa =\max\{\kappa_{{\bar{z}}_{i}}^{\prime }|i=1,\ldots ,k\}$, and $b=\min\{\beta_{{\bar{z}}_{i}}/3|i=1,\ldots ,k\}$. For any z=(p,x)∈Ω there is an index i∈{1,…,k} such that $z\in O_{i}$. Hence the mapping $G_{p}$ is regular at *x* for 0 with the constant *κ* and neighbourhoods ${I\;B}_{a}(x)$ and ${I\;B}_{b}(0)$.

Under the assumption of strong regularity one uses Remark 2.4 (or the strong regularity part of Theorem 5G.3 in [4]).

Remark 2.7 Note that (ii) in Theorem 2.6 is satisfied, in particular, when *σ* has a *point-based approximation on Ω* in the sense of Robinson [6]. Theorem 2.6 yields [5, Lemma 0]. Moreover, given a non-empty subset Ω of a metric space, define the *measure of non-compactness* of Ω by

$$\chi (\Omega ):=\inf\{r>0\;|\;\Omega \subset \Omega_{F}+{I\;B}_{r}(0)\text{ for some finite subset }\Omega_{F}\text{ of }\Omega \}.$$

Then Theorem 2.6 holds provided that χ(Ω) is strictly smaller than the infimum of the reciprocal values of the regularity moduli of the mappings appearing in (i). This statement is a key element in the proof of the non-smooth versions of Robinson and Lyusternik-Graves theorems, cf. [1, Step 1] and [2, Lemma 12].

Next statement guarantees uniform [strong] regularity along continuous paths.

Theorem 2.8 Let *T*>0 be fixed and let (X,∥⋅∥) and (Y,∥⋅∥) be Banach spaces. Consider a set-valued mapping F:X⇉Y with closed graph, a mapping σ:[0,T]×X→Y, and two continuous mappings φ:[0,T]→X and ψ:[0,T]→Y such that

- for each t∈[0,T] the mapping $X∋v⟼G_{t}(v):=\sigma (t,v)+F(v)\subset Y$ is [strongly] regular at φ(t) for ψ(t);
- for each t∈[0,T] and each μ>0 there is δ>0 such that for each *v*, $v^{\prime }\in {I\;B}_{\delta }(\varphi (t))$ and each $t^{\prime }\in {I\;B}_{\delta }(t)$ we have
  $$∥[\sigma (t^{\prime },v^{\prime })-\sigma (t,v^{\prime })]-[\sigma (t^{\prime },v)-\sigma (t,v)]∥\leq \mu ∥v-v^{\prime }∥;$$
- for each x∈X the function σ(⋅,x) is continuous.

Then there are positive constants *a*, *b*, and *κ* such that for each t∈[0,T] the mapping $G_{t}$ is [strongly] regular at φ(t) for ψ(t) with the constant *κ* and neighbourhoods ${I\;B}_{a}(\varphi (t))$ and ${I\;B}_{b}(\psi (t))$.

Proof. Apply Theorem 2.6 with P:=[0,T]×Y, a (compact) set $\Omega :=\underset{t\in [0,T]}{⋃}(t,\psi (t),\varphi (t))$, and σ(p,x):=σ(t,x)−y, p=(t,y)∈P, x∈X.

Clearly, we can replace the interval [0,T] by any compact metric space in the above statement.

## 3. Path-following for differential generalized equations

Consider the DGE (1), with *T*>0, single-valued functions $g:R^{n}\times R^{m}\to R^{n}$ and $f:R^{n}\times R^{m}\to R^{d}$, a set-valued mapping $F:R^{m}⇉R^{d}$, and an initial state $x_{{}_{I}}\in R^{n}$. If it is not clearly indicated otherwise we impose the following:

*Standing assumptions (SA).* Consider the DGE (1) and suppose that *f* and *g* are differentiable functions with a locally Lipschitz continuous derivative, and that *F* has a closed graph. Further, let a pair of functions $\left(\bar{x}(\cdot ),\bar{u}(\cdot )\right)$ be a solution of (1) such that both of them are differentiable on [0,T] and have a Lipschitz continuous derivative on this interval.

For an integer *N*>1, consider the uniform grid $t_{i}:=ih$, i∈{0,1,…,N}, with a step size h:=T/N. Given Δ>0 and points $(e_{i}{)}_{i=0}^{N^{-1}}$ in ${I\;B}_{\Delta h^{2}}(0)$, consider the following iteration

(12) $$\left\{\begin{array}{l} {\tilde{x}}_{i+1}=x_{i}+hg(x_{i},u_{i}),\\ e_{i}\in f({\tilde{x}}_{i+1},u_{i})+\nabla_{u}f({\tilde{x}}_{i+1},u_{i})(u_{i+1}-u_{i})+F(u_{i+1}),\\ x_{i+1}=x_{i}+\frac{h}{2}(g(x_{i},u_{i})+g({\tilde{x}}_{i+1},u_{i+1})), \end{array}\right.$$

with $\left(x_{0},u_{0}\right)$ sufficiently close to $\left(\bar{x}(0),\bar{u}(0)\right)$. The reason for allowing $x_{0}\neq x_{I}$ is that for a given time interval I:=[−T,T], say, one cannot expect that $\bar{u}(\cdot )$ is differentiable on the whole of *I* in general (for example when a geometric constraint represented by the generalized equation is a variational inequality). However, $\bar{u}(\cdot )$ can be piece-wise smooth on *I* and the starting point $x_{0}$ can be viewed as a final iterate obtained by a numerical algorithm on the previous subinterval [−T,0]. In fact, this is the case in our numerical examples. As noted by an anonymous referee the assumptions on the differentiability of $\bar{u}(\cdot )$ could be relaxed by employing the averaged modulus of smoothness to obtain the same estimates when the derivative of $\bar{u}(\cdot )$ is of bounded variation only; also one can consider more general Runge-Kutta approximations as in [7]. However, we prefer to keep the presentation as clear as possible and use a modification of the classical trapezoidal rule [8] in our analysis.

Lemma 3.1 Let φ:[a,b]→R be a function with a Lipschitz continuous derivative on [a,b]. Then there is a constant *m*>0 such that for each $t_{1},$$t_{2}\in [a,b],$ with $t_{1}<t_{2},$ we have

$$\left|\frac{\left(t_{2}-t_{1}\right)}{2}(\varphi (t_{1})+\varphi (t_{2}))-\int_{t_{1}}^{t_{2}}\varphi (t)\;dt\right|\leq m(t_{2}-t_{1}{)}^{3}.$$

Proof. Let ℓ>0 be a Lipschitz constant of $\dot{\varphi }$ on [a,b]. Fix arbitrary $t_{1}$, $t_{2}\in [a,b]$ with $t_{1}<t_{2}$ and let $h:=t_{2}-t_{1}$. Find $\tau_{1}$ and $\tau_{2}$ in $\left[t_{1},t_{2}\right]$ such that $\dot{\varphi }(\tau_{1})=\min_{\tau \in [t_{1},t_{2}]}\dot{\varphi }(\tau )$ and $\dot{\varphi }(\tau_{2})=\max_{\tau \in [t_{1},t_{2}]}\dot{\varphi }(\tau )$. Consider a function $\psi :[t_{1},t_{2}]\to R$ defined by

$$\psi (t):=\varphi (t)-\frac{\dot{\varphi }(\tau_{1})+\dot{\varphi }(\tau_{2})}{2}t,\;t\in [t_{1},t_{2}].$$

For each $t\in [t_{1},t_{2}]$, we have φ˙(τ1)≤a˙φ(t)≤a˙φ(τ2), and consequently

$$\begin{array}{rl} & -\frac{\ell h}{2}\leq -\frac{\ell }{2}|\tau_{1}-\tau_{2}|\leq \frac{1}{2}(\dot{\varphi }(\tau_{1})-\dot{\varphi }(\tau_{2}))\\ & \;\leq \dot{\psi }(t)\leq \frac{1}{2}(\dot{\varphi }(\tau_{2})-\dot{\varphi }(\tau_{1}))\leq \frac{\ell }{2}|\tau_{2}-\tau_{1}|\leq \frac{\ell h}{2}. \end{array}$$

Thus $\max_{\tau \in [t_{1},t_{2}]}|\dot{\psi }(\tau )|\leq \ell h/2$. Basic calculus and the mean value theorem imply that

$$\begin{array}{rl} & \left|\frac{h}{2}(\varphi (t_{1})+\varphi (t_{2}))-\int_{t_{1}}^{t_{2}}\varphi (t)\;dt\right|\\ & =\left|\frac{h}{2}(\psi (t_{1})+\psi (t_{2}))-\int_{t_{1}}^{t_{2}}\psi (t)\;dt\right|\\ & =\left|\int_{t_{1}}^{t_{1}+h/2}[\psi (t_{1})-\psi (t)]\;dt+\int_{t_{1}+h/2}^{t_{2}}[\psi (t_{2})-\psi (t)]\;dt\right|\\ & \leq \max_{\tau \in [t_{1},t_{2}]}|\dot{\psi }(\tau )|\left(\int_{t_{1}}^{t_{1}+h/2}(t-t_{1})\;dt+\int_{t_{1}+h/2}^{t_{2}}(t_{2}-t)\;dt\right)\\ & =\max_{\tau \in [t_{1},t_{2}]}|\dot{\psi }(\tau )|\left(\frac{h^{2}}{8}+\frac{h^{2}}{8}\right)\leq \frac{\ell }{8}h^{3}. \end{array}$$

As ℓ is independent of both $t_{1}$ and $t_{2}$, setting m:=ℓ/8 we finish the proof.

Theorem 3.2 In addition to (SA), suppose that for each t∈[0,T] the mapping

(13) $$R^{m}∋v⟼G_{t}(v):=f(\bar{x}(t),\bar{u}(t))+\nabla_{u}f(\bar{x}(t),\bar{u}(t))(v-\bar{u}(t))+F(v)\subset R^{d}$$

is [strongly] regular at $\bar{u}(t)$ for 0. Then for any Δ>0 there are $N_{0}\in N$ and positive constants *α* and $\bar{d}$ such that for each $N>N_{0},$ each $(x_{0},u_{0})\in {I\;B}_{\Delta h^{2}}(\bar{x}(0))\times {I\;B}_{\Delta h^{2}}(\bar{u}(0)),$ and each $(e_{i}{)}_{i=0}^{N-1}$ in ${I\;B}_{\Delta h^{2}}(0),$ where h:=T/N, there are [uniquely determined] points $(x_{i},u_{i})\in R^{n}\times R^{m},$i∈{1,…,N}, generated by the iteration (12), with the initial point $(x_{0},u_{0}),$ such that $\left(x_{i},u_{i})\in {I\;B}_{\alpha }(\bar{x}(t_{i}))\times {I\;B}_{\alpha }(\bar{u}(t_{i})\right)$ for each i∈{1,…,N} satisfying

(14) $$\max_{0\leq i\leq N}∥x_{i}-\bar{x}(t_{i})∥\leq \bar{d}h^{2}\;\text{and}\;\max_{0\leq i\leq N}∥u_{i}-\bar{u}(t_{i})∥\leq \bar{d}h^{2}.$$

Proof. Let a (continuous) function *σ* be defined by $\sigma (t,v):=f(\bar{x}(t),\bar{u}(t))+\nabla_{u}f(\bar{x}(t),\bar{u}(t))(v-\bar{u}(t))$, $(t,v)\in [0,T]\times R^{m}$. For each t∈[0,T] and each μ>0, the continuity of the function $s⟼\nabla_{u}f(\bar{x}(s),\bar{u}(s))$ at *t* yields a constant δ>0 such that

$$\begin{array}{rl} & ∥\nabla_{u}f(\bar{x}(t^{\prime }),\bar{u}(t^{\prime }))-\nabla_{u}f(\bar{x}(t),\bar{u}(t))∥<\mu \\ & \;\text{whenever }t^{\prime }\in (t-\delta ,t+\delta )\cap [0,T], \end{array}$$

consequently, for any such $t^{\prime }$ and arbitrary *v*, $v^{\prime }\in R^{m}$ we have

$$\begin{array}{rl} & ∥[\sigma (t^{\prime },v^{\prime })-\sigma (t,v^{\prime })]-[\sigma (t^{\prime },v)-\sigma (t,v)]∥\\ & =∥[\nabla_{u}f(\bar{x}(t^{\prime }),\bar{u}(t^{\prime }))-\nabla_{u}f(\bar{x}(t),\bar{u}(t))](v^{\prime }-v)∥\\ & \leq \mu ∥v-v^{\prime }∥. \end{array}$$

Theorem 2.8, with $\varphi :=\bar{u}(\cdot )$ and ψ≡0, yields positive constants *a*, *b*, and *κ* such that for each t∈[0,T] the mapping $G_{t}$ is [strongly] regular at $\bar{u}(t)$ for 0 with the constant *κ* and neighbourhoods ${I\;B}_{a}(\bar{u}(t))$ and ${I\;B}_{b}(0)$. Find $\ell_{1}>0$ such that both $\bar{x}(\cdot )$ and $\bar{u}(\cdot )$ are Lipschitz continuous on [0,T] with the constant $\ell_{1}$. Let *r*>0 be such that $\bar{x}([0,T])+a{I\;B}_{R^{n}}\subset r{I\;B}_{R^{n}}$ and $\bar{u}([0,T])+a{I\;B}_{R^{m}}\subset r{I\;B}_{R^{m}}$. Pick $\ell_{2}>0$ such that *f*, *g*, and $\nabla_{u}f$ are Lipschitz continuous on the (compact) set $r{I\;B}_{R^{n}}\times r{I\;B}_{R^{m}}$. Let

(15) $$\kappa^{\prime }:=2\kappa ,\;\mu :=1/(3\kappa ),\;\text{and}\;\ell :=\max\{1,\ell_{1},\ell_{2}\}.$$

By the basic calculus, for every *u*, $u^{\prime }\in r{I\;B}_{R^{m}}$ and every $x\in r{I\;B}_{R^{n}}$, we have

(16) $$∥f(x,u^{\prime })-f(x,u)-\nabla_{u}f(x,u)(u^{\prime }-u)∥\leq \frac{\ell }{2}∥u^{\prime }-u{∥}^{2}.$$

Let

(17) α:=min{1,a/2,1/(6ℓκ),a/(16κℓ),3κb/(20κℓ+1)}andβ:=2ℓα.

We show the following claim: *For any $\left(t,u,x,y)\in [0,T]\times {I\;B}_{\alpha }(\bar{u}(t))\times {I\;B}_{\alpha }(\bar{x}(t))\times {I\;B}_{\beta }(0\right)$, there is a [unique] point $w\in {I\;B}_{\alpha }(\bar{u}(t))$ such that $y\in f(x,u)+\nabla_{u}f(x,u)(w-u)+F(w)$ and*

$$∥w-\bar{u}(t)∥\leq \kappa^{\prime }\ell (∥x-\bar{x}(t)∥+∥u-\bar{u}(t){∥}^{2}+∥y∥).$$

To prove this, fix any such (t,u,x,y) and consider a function $\varphi :R^{m}\to R^{d}$ defined by

$$\begin{array}{rl} & \varphi (v):=f(x,u)+\nabla_{u}f(x,u)(v-u)-f(\bar{x}(t),\bar{u}(t))\\ & \;-\nabla_{u}f(\bar{x}(t),\bar{u}(t))(v-\bar{u}(t)),\;v\in R^{m}. \end{array}$$

We are going to use Theorem 2.3 (with $G:=G_{t}$ and g:=φ). Note that $G_{t}$ has closed graph. Clearly (15) implies κμ<1 and $\kappa^{\prime }>3\kappa /2=\kappa /(1-\mu \kappa )$. We also get

$$2\kappa^{\prime }\beta +\alpha =(8\kappa \ell )\alpha +\alpha \leq a/2+a/2=a,$$

and, consequently, we obtain that

$$\mu (2\kappa^{\prime }\beta +\alpha )+2\beta =\frac{8\kappa \ell \alpha +\alpha }{3\kappa }+4\alpha \ell =\alpha \frac{20\kappa \ell +1}{3\kappa }\leq b.$$

As $u\in {I\;B}_{\alpha }(\bar{u}(t))\subset {I\;B}_{a}(\bar{u}(t))\subset r{I\;B}_{R^{m}}$ and $x\in {I\;B}_{\alpha }(\bar{x}(t))\subset {I\;B}_{a}(\bar{x}(t))\subset r{I\;B}_{R^{n}}$, by (16) we get

(18) $$\begin{array}{rl} ∥\varphi (\bar{u}(t))∥ & =∥f(\bar{x}(t),\bar{u}(t))-f(x,u)-\nabla_{u}f(x,u)(\bar{u}(t)-u)∥\\ & \leq ∥f(\bar{x}(t),\bar{u}(t))\\ & \;-f(x,\bar{u}(t))∥+∥f(x,\bar{u}(t))-f(x,u)-\nabla_{u}f(x,u)(\bar{u}(t)-u)∥\\ & \leq \ell ∥\bar{x}(t)-x∥+\frac{\ell }{2}∥\bar{u}(t)-u{∥}^{2}<\ell \alpha +\ell \alpha^{2}\leq 2\ell \alpha =\beta . \end{array}$$

Since 2ℓα≤1/(3κ)=μ, for arbitrary *v*, $v^{\prime }\in R^{m}$, we have

$$\begin{array}{rl} ∥\varphi (v)-\varphi (v^{\prime })∥ & =∥(\nabla_{u}f(x,u)-\nabla_{u}f(\bar{x}(t),\bar{u}(t))(v-v^{\prime })∥\\ & \leq \ell (∥x-\bar{x}(t)∥+∥u-\bar{u}(t)∥)∥v-v^{\prime }∥\\ & \;\leq 2\ell \alpha ∥v-v^{\prime }∥\leq \mu ∥v-v^{\prime }∥. \end{array}$$

Moreover, observing that $\varphi +G_{t}=f(x,u)+\nabla_{u}f(x,u)(\cdot -u)+F$, we get

$$\begin{array}{rl} \varphi (\bar{u}(t)) & =f(x,u)+\nabla_{u}f(x,u)(\bar{u}(t)-u)-f(\bar{x}(t),\bar{u}(t))\\ & \in f(x,u)+\nabla_{u}f(x,u)(\bar{u}(t)-u)+F(\bar{u}(t))=(\varphi +G_{t})(\bar{u}(t)). \end{array}$$

Hence $\bar{u}(t)\in (\varphi +G_{t}{)}^{-1}(\varphi (\bar{u}(t)))$ and $\varphi (\bar{u}(t))\in {I\;B}_{\beta }(0)$. Remembering that $y\in {I\;B}_{\beta }(0)$. Theorem 2.3 implies that there is $w\in (\varphi +G_{t}{)}^{-1}(y)$ such that $∥w-\bar{u}(t)∥\leq \kappa^{\prime }∥y-\varphi (\bar{u}(t))∥$. Then $y\in f(x,u)+\nabla_{u}f(x,u)(w-u)+F(w)$ and (18) implies that

$$∥w-\bar{u}(t)∥\leq \kappa^{\prime }(∥y∥+\ell ∥x-\bar{x}(t)∥+\ell ∥u-\bar{u}(t){∥}^{2}),$$

which proves the claim because ℓ≥1.

Use Lemma 3.1 to find *m*>0 such that for each $\tau_{1}$, $\tau_{2}\in [0,T]$, with $\tau_{1}<\tau_{2}$, we have

(19) $$\begin{array}{rl} & ∥\frac{\left(\tau_{2}-\tau_{1}\right)}{2}(g(\bar{x}(\tau_{1}),\bar{u}(\tau_{1}))+g(\bar{x}(\tau_{2}),\bar{u}(\tau_{2})))-\int_{\tau_{1}}^{\tau_{2}}g(\bar{x}(t),\bar{u}(t))\;dt∥\\ & \;\leq m(\tau_{2}-\tau_{1}{)}^{3}. \end{array}$$

Pick an arbitrary Δ>0. Let

$$q:=\max\{4\ell^{2},\Delta ,\kappa^{\prime }\ell ,T^{2},m\},\;\lambda :=4q^{3},\;\text{and}\;\bar{d}:=q(T\lambda e^{T\lambda }+4q).$$

Choose $N_{0}\in N$ such that $2\bar{d}<N_{0}$ and $qT\leq N_{0}\min\{\alpha ,\beta \}$. Fix any $N>N_{0}$ and let h:=T/N. Then

(20) $$h<\frac{T}{N_{0}}\leq \frac{\sqrt{q}}{N_{0}}<\frac{\sqrt{q}}{2\bar{d}}<\frac{1}{2}\;\text{and}\;h\leq qh<q\frac{T}{N_{0}}\leq \min\{\alpha ,\beta \}.$$

Let $\left(x_{0},u_{0})\in {I\;B}_{\Delta h^{2}}(\bar{x}(0))\times {I\;B}_{\Delta h^{2}}(\bar{u}(0)\right)$ and $(e_{i}{)}_{i=0}^{N-1}$ in ${I\;B}_{\Delta h^{2}}(0)$ be arbitrary. For each i∈{0,1,…,N}, let $t_{i}:=ih$ and $c_{i}:=\lambda ie^{\lambda ih}$. Since q≥Δ, we have

$$\begin{array}{rl} & ∥x_{0}-\bar{x}(0)∥\leq qh^{2}=(c_{0}h+q)h^{2}\;\text{and}\\ & \;\;∥u_{0}-\bar{u}(0)∥\leq qh^{2}<q(c_{0}h+4q)h^{2}. \end{array}$$

As $qh^{2}<qh/2<\alpha /2$ we have $\left(x_{0},u_{0})\in {I\;B}_{\alpha }(\bar{x}(t_{0}))\times {I\;B}_{\alpha }(\bar{u}(t_{0})\right)$. We proceed by induction. Suppose that for some i∈{0,1,…,N−1} a point $\left(x_{i},u_{i})\in {I\;B}_{\alpha }(\bar{x}(t_{i}))\times {I\;B}_{\alpha }(\bar{u}(t_{i})\right)$ verifies

(21) $$∥x_{i}-\bar{x}(t_{i})∥\leq (c_{i}h+q)h^{2}\;\text{and}\;∥u_{i}-\bar{u}(t_{i})∥\leq q(c_{i}h+4q)h^{2}.$$

We will show that there are [uniquely determined] points ${\tilde{x}}_{i+1}$, $x_{i+1}\in {I\;B}_{\alpha }(\bar{x}(t_{i+1}))$ and $u_{i+1}\in {I\;B}_{\alpha }(\bar{u}(t_{i+1}))$ satisfying (12) such that (21) holds for *i*:=*i*+1.

Let ${\tilde{x}}_{i+1}$ be defined by the first equality in (12). Clearly, for any $s\in [t_{i},t_{i+1}]$, we have

(22) $$\begin{array}{rl} & ∥g(x_{i},u_{i})-g(\bar{x}(s),\bar{u}(s))∥\\ & \;\leq \ell (∥x_{i}-\bar{x}(s)∥+∥u_{i}-\bar{u}(s)∥)\\ & \leq \ell (∥x_{i}-\bar{x}(t_{i})∥+\ell (s-t_{i})+∥u_{i}-\bar{u}(t_{i})∥+\ell (s-t_{i}))\\ & =\ell (∥x_{i}-\bar{x}(t_{i})∥+∥u_{i}-\bar{u}(t_{i})∥)+2\ell^{2}(s-t_{i}). \end{array}$$

As $c_{i}h<T\lambda e^{T\lambda }$ and $\ell \bar{d}h<q/4$, using (22) and (20) we get

(23) $$\begin{array}{rl} ∥{\tilde{x}}_{i+1}-\bar{x}(t_{i+1})∥ & =∥x_{i}+hg(x_{i},u_{i})-\bar{x}(t_{i})-\int_{t_{i}}^{t_{i+1}}g(\bar{x}(s),\bar{u}(s))\;ds∥\\ & \leq ∥x_{i}-\bar{x}(t_{i})∥+\int_{t_{i}}^{t_{i+1}}∥g(x_{i},u_{i})-g(\bar{x}(s),\bar{u}(s))∥\;ds\\ & \leq ∥x_{i}-\bar{x}(t_{i})∥+\ell h(∥x_{i}-\bar{x}(t_{i})∥+∥u_{i}-\bar{u}(t_{i})∥)+\ell^{2}h^{2}\\ & =(1+\ell h)∥x_{i}-\bar{x}(t_{i})∥+\ell h∥u_{i}-\bar{u}(t_{i})∥+\ell^{2}h^{2}\\ & \leq (1+\ell h)(c_{i}h+q)h^{2}+\ell \bar{d}h^{3}+\ell^{2}h^{2}\\ & =\left(c_{i}h+\ell (c_{i}h+q)h+q+\ell \bar{d}h+\ell^{2}\right)h^{2}\\ & <\left(c_{i}h+\ell \bar{d}h+q+\ell \bar{d}h+q/4\right)h^{2}\\ & <\left(c_{i}h+q/4+q+q/4+q/4\right)h^{2}\\ & <\left(c_{i}h+2q\right)h^{2}<(\bar{d}/q)h^{2}=h(\bar{d}h)/q<h/2<\alpha /2. \end{array}$$

In particular ${\tilde{x}}_{i+1}\in {I\;B}_{\alpha }(\bar{x}(t_{i+1}))$. Remembering that $c_{i}h<T\lambda e^{T\lambda }$, (21) and (20) yield that

(24) $$\begin{array}{rl} ∥u_{i}-\bar{u}(t_{i+1})∥ & \leq ∥u_{i}-\bar{u}(t_{i})∥+∥\bar{u}(t_{i})-\bar{u}(t_{i+1})∥<q(T\lambda e^{T\lambda }+4q)h^{2}+\ell h\\ & =(\bar{d}h)h+\ell h<\sqrt{q}h<\alpha . \end{array}$$

Clearly, $e_{i}\in {I\;B}_{\beta }(0)$. The claim with $t:=t_{i+1}$, $y:=e_{i}$, $x:={\tilde{x}}_{i+1}$, and $u:=u_{i}$ together with (23), (24), and (20) yields a [unique] point $u_{i+1}\in {I\;B}_{\alpha }(\bar{u}(t_{i+1}))$ such that

$$e_{i}\in f({\tilde{x}}_{i+1},u_{i})+\nabla_{u}f({\tilde{x}}_{i+1},u_{i})(u_{i+1}-u_{i})+F(u_{i+1})$$

satisfying

(25) $$\begin{array}{rl} ∥u_{i+1}-\bar{u}(t_{i+1})∥ & \leq q(∥{\tilde{x}}_{i+1}-\bar{x}(t_{i+1})∥+∥u_{i}-\bar{u}(t_{i+1}){∥}^{2}+∥e_{i}∥)\\ & <q\left(c_{i}h+2q+q+\Delta \right)h^{2}\leq q(c_{i}h+4q)h^{2}. \end{array}$$

As $c_{i}<c_{i+1}$, we obtain the latter estimate in (21) with *i*:=*i*+1. Let $x_{i+1}$ be defined by the last equality in (12). Now (19), (21), (23), (25), and (20) imply that

$$\begin{array}{rl} & ∥x_{i+1}-\bar{x}(t_{i+1})∥\\ & =∥x_{i}+\frac{h}{2}(g(x_{i},u_{i})+g({\tilde{x}}_{i+1},u_{i+1}))-\bar{x}(t_{i})-\int_{t_{i}}^{t_{i+1}}g(\bar{x}(s),\bar{u}(s))\;ds∥\\ & \leq ∥x_{i}-\bar{x}(t_{i})∥+mh^{3}+\frac{h}{2}∥g(x_{i},u_{i})+g({\tilde{x}}_{i+1},u_{i+1})\\ & \;-g(\bar{x}(t_{i}),\bar{u}(t_{i}))-g(\bar{x}(t_{i+1}),\bar{u}(t_{i+1}))∥\\ & \leq (c_{i}h+q)h^{2}+mh^{3}+\frac{\ell h}{2}(∥x_{i}-\bar{x}(t_{i})∥\\ & \;+∥u_{i}-\bar{u}(t_{i})∥+∥{\tilde{x}}_{i+1}-\bar{x}(t_{i+1})∥+∥u_{i+1}-\bar{u}(t_{i+1})∥)\\ & \leq (c_{i}+m)h^{3}+qh^{2}\\ & \;+\frac{\ell h}{2}((c_{i}h+q)h^{2}+q(c_{i}h+4q)h^{2}+(c_{i}h+2q)h^{2}+q(c_{i}h+4q)h^{2})\\ & <(c_{i}+q)h^{3}+\frac{h^{3}}{4}(q(c_{i}h+q)+q^{2}(c_{i}h+4q)\\ & \;+q(c_{i}h+2q)+q^{2}(c_{i}h+4q))+qh^{2}\\ & =c_{i}(1+(q+q^{2})h/2)h^{3}+(q+3q^{2}/4+2q^{3})h^{3}\\ & \;+qh^{2}<c_{i}(1+4q^{3}h)h^{3}+4q^{3}h^{3}+qh^{2}\\ & =c_{i}(1+\lambda h)h^{3}+\lambda h^{3}+qh^{2}\leq \lambda ie^{\lambda (i+1)h}h^{3}+\lambda e^{\lambda (i+1)h}h^{3}+qh^{2}\\ & =\lambda (i+1)e^{\lambda (i+1)h}h^{3}+qh^{2}=(c_{i+1}h+q)h^{2}. \end{array}$$

The first estimate in (21) with *i*:=*i*+1 is proved. Since $(c_{i+1}h+q)h^{2}<\bar{d}h^{2}<qh/2<\alpha /2$, we have $x_{i+1}\in {I\;B}_{\alpha }(\bar{x}(t_{i+1}))$. The induction step is complete and so is the proof by noting that for each i∈{0,1,…,N} we have $c_{i}h\leq T\lambda e^{T\lambda }$.

If $\bar{u}(\cdot )$ is only Lipschitz continuous on [0,T], one can consider the following iteration:

(26) $$\left\{\begin{array}{l} x_{i+1}=x_{i}+hg(x_{i},u_{i}),\\ e_{i}\in f(x_{i+1},u_{i})+\nabla_{u}f(x_{i+1},u_{i})(u_{i+1}-u_{i})+F(u_{i+1}). \end{array}\right.$$

Using a similar technique as in the proof of Theorem 3.2 we obtain:

Theorem 3.3 Consider the DGE (1) and suppose that *f* and *g* are differentiable functions with a locally Lipschitz continuous derivative, and that *F* has a closed graph. Let a pair of functions $\left(\bar{x}(\cdot ),\bar{u}(\cdot )\right)$ be a solution of (1) such that both $\bar{x}(\cdot )$ and $\bar{u}(\cdot )$ are Lipschitz continuous on [0,T]. Suppose that for each t∈[0,T] the mapping $G_{t}$ in (13) is [strongly] regular at $\bar{u}(t)$ for 0. Then for any Δ>0 there are $N_{0}\in N$ and positive constants *α* and $\bar{d}$ such that for each $N>N_{0}$, each $(x_{0},u_{0})\in {I\;B}_{\Delta h}(\bar{x}(0))\times {I\;B}_{\Delta h}(\bar{u}(0)),$ and each $(e_{i}{)}_{i=0}^{N-1}$ in ${I\;B}_{\Delta h}(0),$ where h:=T/N, there are [uniquely determined] points $(x_{i},u_{i})\in R^{n}\times R^{m},$i∈{1,…,N}, generated by the iteration (26), with the initial point $(x_{0},u_{0}),$ such that $\left(x_{i},u_{i})\in {I\;B}_{\alpha }(\bar{x}(t_{i}))\times {I\;B}_{\alpha }(\bar{u}(t_{i})\right)$ for each i∈{1,…,N} satisfying

(27) $$\max_{0\leq i\leq N}∥x_{i}-\bar{x}(t_{i})∥\leq \bar{d}h\;\text{and}\;\max_{0\leq i\leq N}∥u_{i}-\bar{u}(t_{i})∥\leq \bar{d}h.$$

The above statement is a slight extension of [3, Theorem 5.1]. Next, we discuss two basic examples from engineering, which can be formulated either as a DGE or an ODE with a Lipschitz continuous right-hand side. We compare schemes (12) and (26) with the method *ODE45* which is used with the absolute error tolerance $10^{-12}$ to get a reference solution trajectory. All simulations are performed in MATLAB.

**Figure 1.**
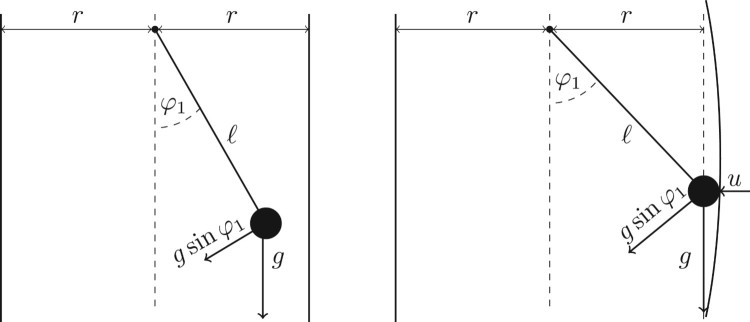
Mechanical model from Example 3.4.

Example 3.4 Consider a particle of mass *m*>0 connected by a rigid, weightless rod of length ℓ>0 to a base by means of a pin joint that can rotate in a plane due to gravity. In addition, the pendulum can have a contact with two walls made of a very flexible material which are at a distance *r*>0 from a pin joint. The contact force acting on the mass at time *t* is denoted by u(t); and $\varphi_{1}(t)$ and $\varphi_{2}(t)$ denote the angular displacement and the angular velocity at time *t*, respectively (see Figure 1).

The equations of motion of the system are

$$\left\{\begin{array}{l} {\dot{\varphi }}_{1}(t)=\varphi_{2}(t),\\ {\dot{\varphi }}_{2}(t)=-\frac{g}{\ell }\sin\varphi_{1}(t)-\frac{1}{\ell m}H(\varphi_{1}(t)),\\ \varphi_{1}(0)=\gamma_{1},\;\varphi_{2}(0)=\gamma_{2}, \end{array}\right.\;\text{for all }t\in [0,T],$$

with given initial conditions $\gamma_{1}$, $\gamma_{2}\in R$, a gravitational acceleration *g*=9.81 ms^−2^, and $u(t)=H(\varphi_{1}(t))$ describing the dependence of the contact force on the angular displacement. We assume that

$$H(\varphi )=\left\{\begin{array}{ll} \mathrm{argsinh}\left(\varphi -\arcsin\left(r/\ell \right)\right) & \text{for }\varphi >\arcsin\left(\ell /r\right),\\ \mathrm{argsinh}\left(\varphi +\arcsin\left(r/\ell \right)\right) & \text{for }\varphi <-\arcsin\left(\ell /r\right),\\ 0 & \text{otherwise.} \end{array}\right.$$

**Figure 2.**
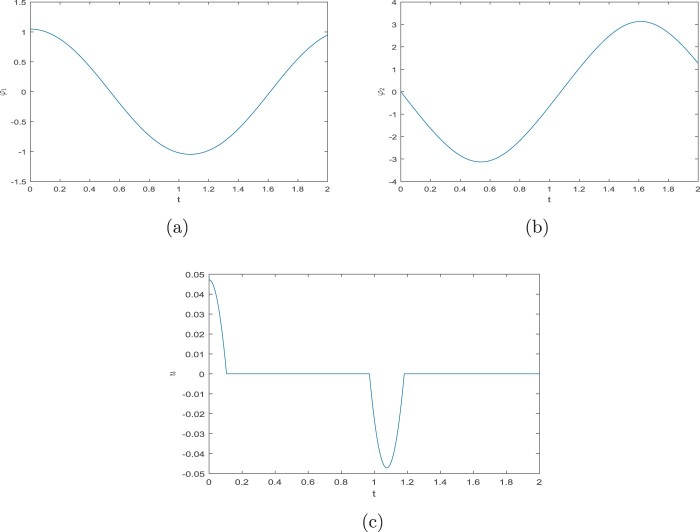
The solution from Example 3.4. (a) The first component $\varphi_{1}$, (b) The second component $\varphi_{2}$ and (c) The third component *u*.

**Figure 3.**
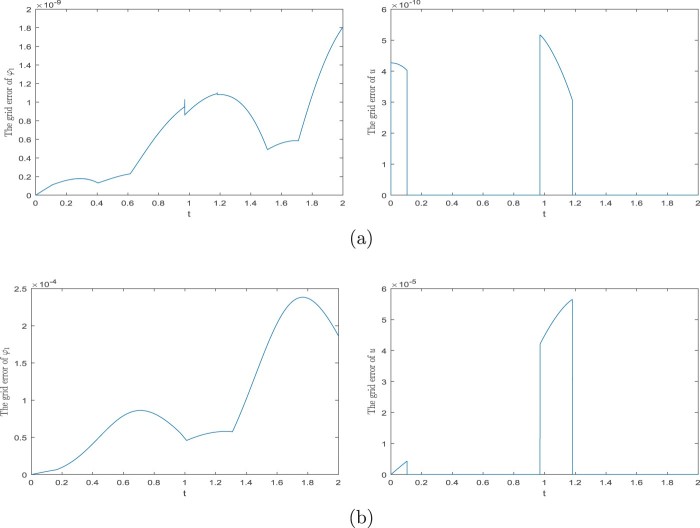
Errors of the solution from Example 3.4. (a) Grid errors of the scheme (12) and (b) Grid errors of the scheme (26).

The corresponding DGE has form

$$\left\{\begin{array}{l} {\dot{\varphi }}_{1}(t)=\varphi_{2}(t),\\ {\dot{\varphi }}_{2}(t)=-\frac{g}{\ell }\sin\varphi_{1}(t)-\frac{1}{\ell m}u(t),\\ 0\in -\varphi_{1}(t)+\sinh u(t)+\arcsin\left(r/\ell \right)\partial |\cdot |(u(t)),\\ \varphi_{1}(0)=\gamma_{1},\;\varphi_{2}(0)=\gamma_{2}, \end{array}\right.\;\text{for all }t\in [0,T],$$

where ∂ denotes a subdifferential in the sense of convex analysis. The solution for ℓ=m:=1,r:=sin1, $T:=2,\gamma_{1}=\pi /3$, and $\gamma_{2}=0$ is in Figure 2. The grid errors with respect to the solution obtained by *ODE45* are in Figure 3. For both the schemes (12) and (26), we use the discretion step $h=10^{-5}$ and $e_{i}=0$, i∈{0,1,…,N−1}.

**Figure 4.**
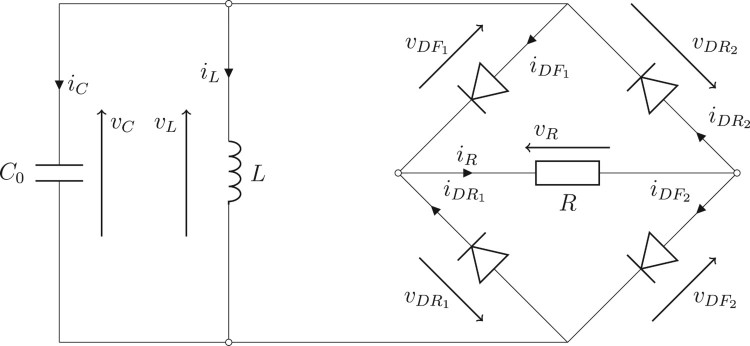
The circuit from Example 3.5.

Example 3.5 Consider a circuit in Figure 4 involving the four-diodes bridge full-wave rectifier, a resistor with a resistance *R*>0, a capacitor with the capacitance $C_{0}>0$ and an inductor with the inductance *L*>0. Denote $v_{C}$ a voltage across the capacitor, $i_{C}$ a current through the capacitor, $i_{L}$ a current through the inductor, $i_{DF1},i_{DF2},i_{DR1},i_{DR2}$ currents through the diodes, and $v_{DF1}$, $v_{DF2}$, $v_{DR1}$, $v_{DR2}$ voltages across the diodes, respectively. Using the Kirchhoff's laws, this problem can be described as a particular DGE (see [9]) called a *differential linear complementarity problem (system)* in the form

(28) $$\left\{\begin{array}{l} \dot{x}(t)=Ax(t)+Bu(t),\\ 0\leq Cx(t)+Du(t)⊥u(t)\geq 0,\\ x(0)=x_{{}_{I}}, \end{array}\right.\;t\in [0,T],$$

where

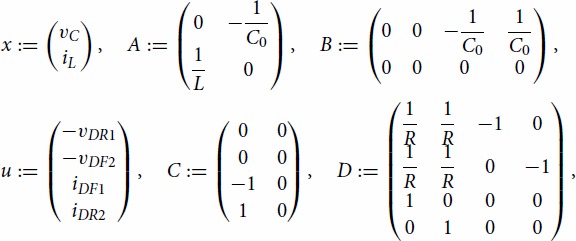

the symbol ⊥ denotes a complementarity relation, and inequalities in $R^{4}$ are understood coordinate-wise. From (28) we have $v_{DR1}(t)=-\max\{v_{C}(t),0\}$, $v_{DF2}(t)=-\max\{-v_{C}(t),0\}$, $i_{DF1}(t)=1/R\max\{v_{C}(t),0\}$, and $i_{DR2}(t)=1/R\max\{-v_{C}(t),0\}$ for each t∈[0,T]. Hence the problem is equivalent to the system of ordinary differential equations, in the form

$$\dot{x}(t)=Ax(t)+Bu(t),\;t\in [0,T],\;\text{and}\;x(0)=x_{{}_{I}}.$$

**Figure 5.**
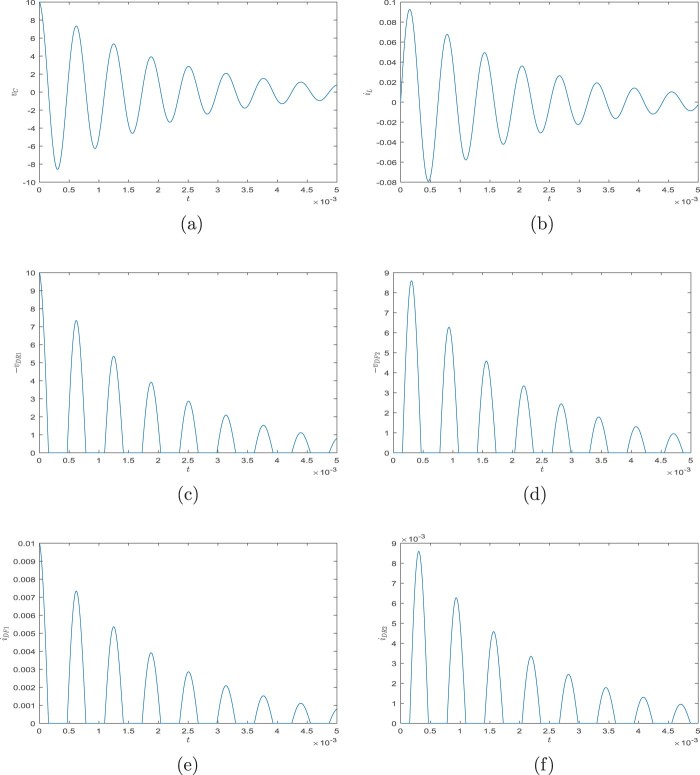
Graphs of the solution from Example 3.5. (a) The first component of x(⋅), (b) The second component of x(⋅), (c) The first component of u(⋅), (d) The second component of u(⋅), (e) The third component of u(⋅) and (f) The fourth component of u(⋅).

**Figure 6.**
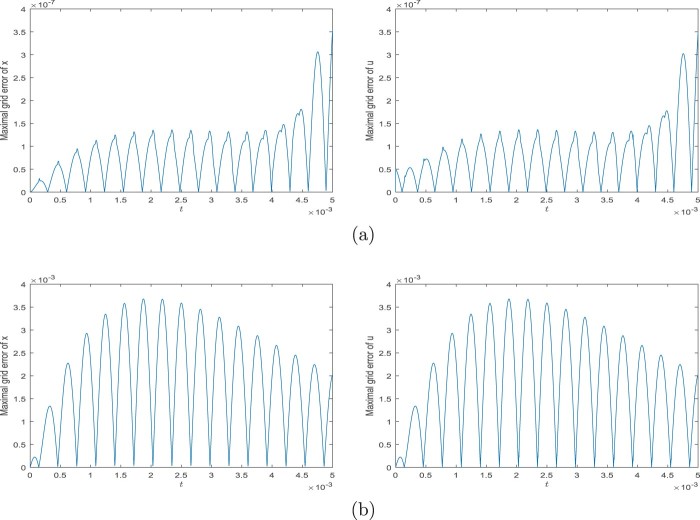
Errors of the solution from Example 3.5. (a) Maximal grid error of the scheme (12) and (b) Maximal grid error of the scheme (26).

For the simulation we use library *LCP*^1^ and assume that $C_{0}:=10^{-6}$, *L*:=0.01, *R*:=1000, *T*:=0.005, and $x_{{}_{I}}:=[10,0]$. For both the schemes (12) and (26), we use the discretion step $h=10^{-8}$ and $e_{i}=0$, i∈{0,1,…,N−1}. Graphs of solution components are in Figure 5 while grid errors are in Figure 6. We note that the maximal grid error means the biggest error of elements of *u* or *x* at the points of the grid.

To conclude this section, let us point out that a similar technique, can be used also in the case of a *parametric generalized equation*, which is a problem for a fixed function $p:[0,T]\to R^{n}$, find a function $z:[0,T]\to R^{n}$ such that

(29) p(t)∈f(z(t))+F(z(t))for all t∈[0,T],

where a constant *T*>0, a function $f:R^{n}\to R^{n}$ and a set-valued mapping $F:R^{n}⇉R^{n}$ are given. This problem can be used, for example, for modelling static problems from electronics, that is, when no capacitors and inductors appear in the circuit [10–13].

For an integer *N*>1, define the uniform grid $t_{i}:=ih$, i∈{0,1,…,N}, with a step size h:=T/N. Given Δ>0 and points $(e_{i}{)}_{i=0}^{N^{-1}}$ in ${I\;B}_{\Delta h^{2}}(p(t_{i+1}))$, we study a predictor-corrector scheme in the form

(30) $$\left\{\begin{array}{l} e_{i}\in f(z_{i})+\nabla f(z_{i})(v_{i+1}-z_{i})+F(v_{i+1}),\\ p(t_{i+1})\in f(v_{i+1})+\nabla f(v_{i+1})(z_{i+1}-v_{i+1})+F(z_{i+1}), \end{array}\right.$$

where $z_{0}$ is sufficiently close to the exact solution of (29) at time *t*:=0. Uniform regularity along a continuous path was used in [14] to obtain the following extension of the main result from [15].

Theorem 3.6 Let $\bar{z}:[0,T]\to R^{n}$ be a Lipschitz continuous solution of the problem (29), where $p:[0,T]\to R^{n}$ is Lipschitz continuous, $f:R^{n}\to R^{n}$ has a locally Lipschitz continuous derivative on whole of $R^{n},$ and $F:R^{n}⇉R^{n}$ has a closed graph. Suppose that for each t∈[0,T] the mapping

$$R^{n}∋v⟼G_{t}(v):=f(\bar{z}(t))+\nabla f(\bar{z}(t))(v-\bar{z}(t))+F(v)\subset R^{n}$$

is [strongly] regular at $\bar{z}(t)$ for p(t). Then there is α>0 such that for any Δ>0 there are constants $N_{0}\in N$ and *c*>0 such that for each $N>N_{0}$ and each $z_{0}\in {I\;B}_{\Delta h^{4}}(\bar{z}(t_{0})),$ where h:=T/N, there are [uniquely determined] points $(z_{i}{)}_{i=1}^{N}$ generated by the iteration (30), with the initial point $z_{0}$ and arbitrarily chosen points $(e_{i}{)}_{i=0}^{N-1}$ in ${I\;B}_{\Delta h^{2}}(p(t_{i+1})),$ such that $z_{i}\in {I\;B}_{\alpha }(\bar{z}(t_{i}))$ for each i∈{0,…,N} and

(31) $$\max_{0\leq i\leq N}∥z_{i}-\bar{z}(t_{i})∥\leq ch^{4}.$$

The point $e_{i}$ appearing in (30) can be interpreted as a sufficiently precise prediction at time $t_{i}$ of the (possibly unknown) value of $p(t_{i+1})$. Then we wait until the precise value of $p(t_{i+1})$ is known and compute a correction $z_{i+1}$. On the other hand, taking $e_{i}:=p(t_{i})+hp^{\prime }(t_{i})$, i∈{0,1,…,N−1}, we have $∥e_{i}-p(t_{i+1})∥\leq \Delta h^{2}$ provided that $p^{\prime }(\cdot )$ exists and is Lipschitz on [0,T] with the constant 2Δ. Hence the algorithm proposed in [4, Section 6G] is a particular case of (30). Finally, instead of $p(t_{i+1})$ in the latter inclusion of (30) one can take any ${\tilde{e}}_{i}\in {I\;B}_{\Delta h^{4}}(p(t_{i+1}))$, that is, the corrector step can be done via an inexact method (which is always the case in practice). Finally, let us note that sufficient conditions (of different type) guaranteeing the existence of a Lipschitz continuous solution $\bar{z}(\cdot )$ of (29) can be found either in [14, Theorem 6] or [3, Theorem 11].

## 4. Uniform regularity and regularity in function spaces

In case that the solution trajectory is not continuous (or even defined) on the whole time interval we can derive the following statement.

Theorem 4.1 Let *T*>0 and *S* be a non-empty subset of [0,T]. Consider a pair of bounded functions $\bar{x}:S\to R^{n}$ and $\bar{u}:S\to R^{m}$ such that

$$0\in f(\bar{x}(t),\bar{u}(t))+F(\bar{u}(t))\;\text{for each }t\in S,$$

with a continuous $f:R^{n}\times R^{m}\to R^{d}$ having a continuous derivative $\nabla_{u}f$ and $F:R^{m}⇉R^{d}$ having a closed graph. Let $\Lambda :=\cup_{t\in S}(\bar{x}(t),\bar{u}(t))$ and for each (x,u)∈cl⁡Λ define a mapping

(32) $$R^{m}∋v⟼G_{x,u}(v):=f(x,u)+\nabla_{u}f(x,u)(v-u)+F(v)\subset R^{d}.$$

Then the following statements are equivalent:

- for each (x,u)∈cl⁡Λ the mapping $G_{x,u}$ is [strongly] regular at *u* for 0;
- there are positive constants *a*, *b*, and *κ* such that for each (x,u)∈cl⁡Λ the mapping $G_{x,u}$ is [strongly] regular at *u* for 0 with the constant *κ* and neighbourhoods ${I\;B}_{a}(u)$ and ${I\;B}_{b}(0);$
- there are positive constants *a*, *b*, and *κ* such that for each t∈S the mapping $G_{t}$ in (13) is [strongly] regular at $\bar{u}(t)$ for 0 with the constant *κ* and neighbourhoods ${I\;B}_{a}(\bar{u}(t))$ and ${I\;B}_{b}(0)$.

Proof. Assume that (i) holds. Define a (compact) set $\Omega :=\mathrm{cl}(\cup_{t\in S}(\bar{x}(t),\bar{u}(t),\bar{u}(t)))$ and a (continuous) function $\sigma (x,u,v):=f(x,u)+\nabla_{u}f(x,u)(v-u)$, $(x,u,v)\in R^{n}\times R^{m}\times R^{m}$. Note that (x,u,v)∈Ω if and only if *v*=*u* and (x,u)∈cl⁡Λ. Theorem 2.6 yields positive constants *a*, *b*, and *κ* such that for each (x,u,u)∈Ω the mapping $G_{x,u}$ is [strongly] regular at *u* for 0 with the constant *κ* and neighbourhoods ${I\;B}_{a}(u)$ and ${I\;B}_{b}(0)$. Since $(\bar{x}(t),\bar{u}(t),\bar{u}(t))\in \Omega$ and $G_{t}=G_{\bar{x}(t),\bar{u}(t)}$ for each t∈S, (iii) is proved.

Assume that (iii) holds. Let $\kappa^{\prime }:=2\kappa$ and μ:=1/(3κ). Then κμ<1 and $\kappa^{\prime }>\kappa /(1-\kappa \mu )$. Pick *r*>0 such that $\bar{x}(S)+a{I\;B}_{R^{n}}\subset r{I\;B}_{R^{n}}$ and $\bar{u}(S)+a{I\;B}_{R^{m}}\subset r{I\;B}_{R^{m}}$. As *f* and $\nabla_{u}f$ are continuous, they are uniformly continuous on a compact set $\Omega :=r{I\;B}_{R^{n}}\times r{I\;B}_{R^{m}}$. Find β>0 such that both $2\kappa^{\prime }\beta +\beta <a$ and $\mu (2\kappa^{\prime }\beta +\beta )+2\beta <b$; and also that for each (x,u)∈Ω and each $(x^{\prime },u^{\prime })\in ({I\;B}_{2\kappa^{\prime }\beta +\beta }(x)\times {I\;B}_{2\kappa^{\prime }\beta +\beta }(u))\cap \Omega$ we have

$$\begin{array}{rl} & ∥\nabla_{u}f(x^{\prime },u^{\prime })-\nabla_{u}f(x,u)∥<\mu \;\text{and}\;∥f(x^{\prime },u^{\prime })-f(x,u)\\ & \;-\nabla_{u}f(x^{\prime },u^{\prime })(u^{\prime }-u)∥<\beta . \end{array}$$

Fix any (x,u)∈cl⁡Λ⊂Ω. Then $0\in G_{x,u}(u)$ since *f* is continuous and gph⁡F is closed. Find $\bar{t}\in S$ such that $\left(x,u)\in {I\;B}_{\beta }(\bar{x}(\bar{t}))\times {I\;B}_{\beta }(\bar{u}(\bar{t})\right)$. Then $G_{x,u}=G_{\bar{t}}+g$, with

$$\begin{array}{rl} g(v) & =f(x,u)+\nabla_{u}f(x,u)(v-u)-f(\bar{x}(\bar{t}),\bar{u}(\bar{t}))\\ & \;-\nabla_{u}f(\bar{x}(\bar{t}),\bar{u}(\bar{t}))(v-\bar{u}(\bar{t})),\;v\in R^{m}. \end{array}$$

Then $∥g(\bar{u}(\bar{t}))∥=∥f(x,u)-f(\bar{x}(\bar{t}),\bar{u}(\bar{t}))-\nabla_{u}f(x,u)(u-\bar{u}(\bar{t}))∥<\beta$. Moreover, for any *v*, $v^{\prime }\in R^{m}$ we have $∥g(v)-g(v^{\prime })∥=∥[\nabla_{u}f(x,u)-\nabla_{u}f(\bar{x}(\bar{t}),\bar{u}(\bar{t}))](v-v^{\prime })∥\leq \mu ∥v-v^{\prime }∥$. Applying Theorem 2.3, with α:=β, and using a similar reasoning as in the proof of Theorem 2.6 we conclude that the mapping $G_{x,u}$ is [strongly] regular at *u* for 0 uniformly in (x,u)∈cl⁡Λ. Hence (ii) holds. Clearly, (ii) implies (i).

The above statement is a generalization of [3, Theorem 7], where strong regularity is considered only, because it requests point-wise [strong] regularity on the closure of the range of the solution instead of on the closure of its graph. The function $\bar{x}(\cdot )$ can be either an input signal in a parametric generalized equation (29) or a state trajectory of the DGE (1). In the latter case, $\bar{x}(\cdot )$ is continuous on S=[0,T], so if $\bar{u}(\cdot )$ has closed range, then the uniform [strong] regularity of $G_{t}$ in (13) on *S* is equivalent to its point-wise [strong] regularity on *S*. We also get the following *uniform* version of the Lyusternik-Graves and Robinson theorem which implies [3, Theorem 9] under substantially weaker assumptions.

Theorem 4.2 Let *T*, *S*, $\bar{x}(\cdot ),$$\bar{u}(\cdot ),$*f*, and *F* be as in Theorem 4.1. Then the mapping $G_{t}=f(\bar{x}(t),\cdot )+F$ is [strongly] regular at $\bar{u}(t)$ for 0 uniformly in t∈S if and only if so is the mapping $G_{t}$ in (13).

Proof. Suppose that there are positive constants *a*, *b* and *κ* such that for each t∈S the mapping $G_{t}$ in (13) is [strongly] regular at $\bar{u}(t)$ for 0 with the constant *κ* and neighbourhoods ${I\;B}_{a}(\bar{u}(t))$ and ${I\;B}_{b}(0)$. Let *β*, $\kappa^{\prime }$, *μ*, *r*, Ω be as in the proof of (iii)⇒(ii) in Theorem 4.1. Fix any t∈S. Let $g_{t}(v):=f(\bar{x}(t),v)-f(\bar{x}(t),\bar{u}(t))-\nabla_{u}f(\bar{x}(t),\bar{u}(t))(v-\bar{u}(t))$, $v\in R^{m}$. Then $g_{t}(\bar{u}(t))=0$ and for any *v*, $v^{\prime }\in {I\;B}_{2\kappa^{\prime }\beta +\beta }(\bar{u}(t))$ we have

$$\begin{array}{rl} ∥g_{t}(v)-g_{t}(v^{\prime })∥ & =∥f(\bar{x}(t),v)-f(\bar{x}(t),v^{\prime })-\nabla_{u}f(\bar{x}(t),\bar{u}(t))(v-v^{\prime })∥\\ & =∥\int_{0}^{1}(\nabla_{u}f(\bar{x}(t),v^{\prime }+s(v-v^{\prime }))\\ & \;-\nabla_{u}f(\bar{x}(t),\bar{u}(t)))(v-v^{\prime })\;ds∥\\ & \leq \mu ∥v-v^{\prime }∥. \end{array}$$

As in Theorem 4.1 we conclude that the mapping $G_{t}=g_{t}+G_{t}$ is [strongly] regular at $\bar{u}(t)$ for 0 uniformly in t∈S. The converse implication follows in the same way.

Before continuing we set up notions used later.

*Notation*(N). Let a constant *T*>0, twice differentiable functions $f:R^{n}\times R^{m}\to R^{d}$ and $g:R^{n}\times R^{m}\to R^{n}$, and a closed convex subset $U_{ad}$ of $R^{d}$ be given. Consider the problem (2). The controls u(⋅) are assumed to be in $U:=L^{\infty }([0,T],R^{m})$, the space of essentially bounded and measurable functions on [0,T] with values in $R^{m}$ considered with the norm $∥u(\cdot ){∥}_{\infty }:=\text{ess sup}∥u(\cdot )∥$, u(⋅)∈U. The state trajectories x(⋅) belong to $X:=W_{0}^{1,\infty }([0,T],R^{n})$, the space of Lipschitz continuous functions on [0,T] with values in $R^{n}$ satisfying x(0)=0 equipped with the norm $∥x(\cdot ){∥}_{X}=∥x(\cdot ){∥}_{\infty }+∥\dot{x}(\cdot ){∥}_{\infty }$, x(⋅)∈X. Let V:=X×U, $R:=L^{\infty }([0,T],R^{n})$, $P:=L^{\infty }([0,T],R^{d})$,

$$U_{ad}:=\{u(\cdot )\in U\;|\;u(t)\in U_{ad}\text{ for a.e. }t\in [0,T]\},$$

and W:=R×P. Given a solution $(\bar{x}(\cdot ),\bar{u}(\cdot ))\in V$ of (2) we set $A(t)=\nabla_{x}g(\bar{x}(t),\bar{u}(t))$, $B(t)=\nabla_{u}g(\bar{x}(t),\bar{u}(t))$, $C(t)=\nabla_{x}f(\bar{x}(t),\bar{u}(t))$, $D(t)=\nabla_{u}f(\bar{x}(t),\bar{u}(t))$, and $\bar{f}(t)=f(\bar{x}(t),\bar{u}(t))$ for a.e. t∈[0,T]. Let Φ be the fundamental matrix solution of the linear equation $\dot{z}=A(t)z$, that is, $\frac{d}{dt}\Phi (t,\tau )=A(t)\Phi (t,\tau )$, Φ(τ,τ)=I.

Consider a set-valued mapping H:V⇉W defined by

$$V∋(x(\cdot ),u(\cdot ))⟼H(x(\cdot ),u(\cdot )):=\left(\begin{array}{c} \dot{x}(t)-g(x(t),u(t))\\ f(x(t),u(t))-U_{ad} \end{array}\right)\subset W$$

along with its shifted partial linearization H at $\left(\bar{x}(\cdot ),\bar{u}(\cdot )\right)$ defined for each (z(⋅),v(⋅))∈V by

$$H(z(\cdot ),v(\cdot )):=\left(\begin{array}{c} \dot{z}(t)-A(t)\;z(t)-B(t)\;v(t)\\ \bar{f}(t)+C(t)\;z(t)+D(t)\;v(t)-U_{ad} \end{array}\right)\subset W,$$

a mapping K:U⇉P defined as

$$\begin{array}{rl} K[v(\cdot )](t): & =\bar{f}(t)+C(t)\int_{0}^{t}\Phi (t,\tau )B(\tau )v(\tau )\;d\tau \\ & \;+D(t)\;v(t)-U_{ad},\;v(\cdot )\in U, \end{array}$$

and mappings $G_{t}$, $G_{t}:R^{m}\to R^{d}$, t∈S, defined, respectively, for each $v\in R^{m}$ by

$$G_{t}(v):=f(\bar{x}(t),v)-U_{ad}\;\text{and}\;G_{t}(v):=\bar{f}(t)+D(t)(v-\bar{u}(t))-U_{ad}.$$

Now we are ready to formulate and prove the main result of this section generalizing [3, Theorem 3].

Theorem 4.3 Under the notation (N), the following assertions are equivalent:

- *H* is regular at $\left(\bar{x}(\cdot ),\bar{u}(\cdot )\right)$ for 0;
- H is regular at (0,0) for 0;
- K is regular at 0 for 0;
- there is a subset *S* of [0,T] having full Lebesgue measure such that the mapping $G_{t}$ is regular at $\bar{u}(t)$ for 0 uniformly in t∈S;
- there is a subset *S* of [0,T] having full Lebesgue measure such that the mapping $G_{t}$ is regular at $\bar{u}(t)$ for 0 uniformly in t∈S;
- there is δ>0 such that for every w(⋅)∈P with $∥w(\cdot ){∥}_{\infty }<\delta$ there is v(⋅)∈U with $∥v(\cdot ){∥}_{\infty }\leq 1$ such that
  $$\begin{array}{rl} & \bar{f}(t)+C(t)\int_{0}^{t}\Phi (t,\tau )B(\tau )v(\tau )\;d\tau +D(t)v(t)+w(t)\in U_{ad}\\ & \;\text{for a.e. }t\in [0,T]; \end{array}$$
- there are δ>0 and *r*>0 such that for every w(⋅)∈P with $∥w(\cdot ){∥}_{\infty }<\delta$ there is a pair $(z(\cdot ),v(\cdot ))\in r{I\;B}_{X}\times r{I\;B}_{U}$ such that
  $$\bar{f}(t)+C(t)\;z(t)+D(t)\;v(t)+w(t)\in U_{ad}\;\text{for a.e. }t\in [0,T].$$

Proof. Define a bounded linear mapping Q:R→X by $Q[r(\cdot )](t)=\int_{0}^{t}\Phi (t,\tau )\;r(\tau )\;d\tau$ for t∈[0,T]. Let $\nu :=\max\{∥A(\cdot ){∥}_{\infty },∥B(\cdot ){∥}_{\infty },∥C(\cdot ){∥}_{\infty },∥D(\cdot ){∥}_{\infty },∥\bar{x}(\cdot ){∥}_{\infty },∥\bar{u}(\cdot ){∥}_{\infty }\}$.

Applying the Lyusternik-Graves theorem [4, Theorem 5E.6] and substituting $z(\cdot )=x(\cdot )-\bar{x}(\cdot )$ and $v(\cdot ):=u(\cdot )-\bar{u}(\cdot )$, we obtain that (i)⇔(ii). By Theorem 4.2 we have (iv)⇔(v) because $\bar{x}(\cdot )$ is continuous and $\bar{u}(\cdot )$ is essentially bounded.

To prove that (ii)⇔(iii), note that given r(⋅)∈R, one has that $\dot{z}(t)-A(t)z(t)=r(t)$ for a.e. t∈[0,T] and z(0)=0 if and only if z(t)=Q[r(⋅)](t), t∈[0,T]. This implies that having (r(⋅),p(⋅))∈H(z(⋅),v(⋅)) is the same as having w(t)∈K[v(⋅)](t) for w(t)=p(t)−C(t)Q[r(⋅)](t), that is, we can replace the differential expression in H with the integral one and then drop the variable *z*. Moreover, $∥w(\cdot ){∥}_{\infty }$ is bounded by a quantity proportional to $∥(r(\cdot ),p(\cdot )){∥}_{W}$.

As K has a closed convex graph, (iii)⇔(vi) by Robinson-Ursescu theorem [4, Theorem 5B.4]. If (vi) holds then setting z(t):=Q[B(⋅)v(⋅)](t), t∈[0,T], we get (vii) with r:=max{1,ν∥Q∥}.

Suppose that (vii) holds. We shall establish (v). Pick β>0 such that ${\bar{w}}_{\beta }(\cdot )\equiv (\beta ,\beta ,\ldots ,\beta )\in R^{d}$ has $∥{\bar{w}}_{\beta }(\cdot ){∥}_{\infty }<\delta$. Let $\left\{w_{1},w_{2},\ldots \right\}$ be a countable dense subset of ${I\;B}_{\beta }(0)$. For any i∈N, the function $w_{i}(\cdot )\equiv -w_{i}$ has $∥w_{i}(\cdot ){∥}_{\infty }\leq ∥{\bar{w}}_{\beta }(\cdot ){∥}_{\infty }<\delta$, thus there is a subset $S_{i}$ of [0,T] having a full Lebesgue measure along with a pair $(z_{i}(\cdot ),v_{i}(\cdot ))\in r{I\;B}_{X}\times r{I\;B}_{U}$ such that

$$\bar{f}(t)+C(t)\;z_{i}(t)+D(t)v_{i}(t)-w_{i}\in U_{ad}\;\text{for all }t\in S_{i}.$$

Without any loss of generality assume that $∥z_{i}(t)∥\leq r$ and $∥v_{i}(t)∥\leq r$ whenever $t\in S_{i}$. Then $S:=\cap_{i=1}^{\infty }S_{i}$ has a full Lebesgue measure. Without any loss of generality assume that ∥C(t)∥≤ν and $\bar{u}(t)$ is defined whenever t∈S. Fix any t∈S. Define a mapping $F_{t}(z,v):=\bar{f}(t)+C(t)\;z+D(t)\;v-U_{ad}$, $(z,v)\in R^{n}\times R^{m}$. For every i∈N we have $w_{i}\in F_{t}(r{I\;B}_{R^{n}}\times r{I\;B}_{R^{m}})$. Hence the image of $r{I\;B}_{R^{n}}\times r{I\;B}_{R^{m}}$ under $F_{t}$ (having a closed convex graph) is dense in ${I\;B}_{\beta }(0)$, and consequently applying Robinson-Ursescu theorem [16, Theorem 6.22] we get that $F_{t}$ is regular at (0,0) for 0 with modulus r/β. In particular, the regularity modulus does not depend on the choice of t∈S. Let Λ be the set in Theorem 4.1. Fix any (x,u)∈cl⁡Λ. Let

$$F_{x,u}(z,v):=f(x,u)+\nabla_{x}f(x,u)z+\nabla_{u}f(x,u)v-U_{ad},\;(z,v)\in R^{n}\times R^{m}.$$

Then $0\in F_{x,u}(0,0)$ since *f* is continuous and $U_{ad}$ is closed. Since $\nabla_{x}f$ and $\nabla_{u}f$ are continuous, the uniformity of the regularity moduli of mappings $F_{t}$ and the Lyusternik-Graves theorem imply that $F_{x,u}$ is regular at (0,0) for 0. Thus the mapping $F_{x,u}^{\prime }(z,v):=F_{x,u}(z,v-u)$, $(z,v)\in R^{n}\times R^{m}$, is regular at (0,u) for 0. Since $w\in F_{x,u}^{\prime }(z,v)$ if and only if $w-\nabla_{x}f(x,u)z\in G_{x,u}(v)$, where $G_{x,u}$ is the mapping in (32) with $F\equiv -U_{ad}$, we conclude that $G_{x,u}$ is regular at *u* for 0. Theorem 4.1 implies that (v) holds.

Suppose that (v) holds. We shall establish (ii) and the theorem will be proved. Assume without any loss of generality that

$$\sup\{∥A(t)∥,∥B(t)∥,∥C(t)∥,∥D(t)∥,∥\bar{u}(t)∥,∥\bar{x}(t)∥\}\leq \nu \;\text{for each }t\in S.$$

Theorem 4.1 implies that there are positive constants *a*, *b* and *κ* such that for any (x,u)∈cl⁡Λ, with $\Lambda :=\cup_{t\in S}(\bar{x}(t),\bar{u}(t))$, the mapping

$$G_{x,u}(v):=f(x,u)+\nabla_{u}f(x,u)(v-u)-U_{ad},\;v\in R^{m},$$

is regular at *u* for 0 with the constant *κ* and neighbourhoods ${I\;B}_{a}(u)$ and ${I\;B}_{b}(0)$. Pick ℓ>κ and then β∈(0,min{a/ℓ,b}/2). Let $\Omega :={I\;B}_{\beta }(0)\times \mathrm{cl}\Lambda$ and consider a mapping

$$\Omega ∋(y,x,u)⟼\Sigma (y,x,u):=G_{x,u}^{-1}(y)\cap {I\;B}_{\ell ∥y∥}(u)\subset R^{m}.$$

Given w:=(y,x,u)∈Ω, the regularity of $G_{x,u}$ at *u* for 0 implies that there is $v\in G_{x,u}^{-1}(y)$ such that ∥u−v∥≤ℓ∥y∥ (with the strict inequality when y≠0), which means that v∈Σ(w). The set $U_{ad}$ is both closed and convex hence so is $G_{x,u}^{-1}(y)$, and consequently also Σ(w). We showed that dom⁡Σ=Ω and Σ has closed convex values.

Since $\Sigma (w)\subset {I\;B}_{\ell ∥y∥}(u)$ for any w∈Ω and $\Sigma (0,\bar{x},\bar{u})=\{\bar{u}\}$ for each $(\bar{x},\bar{u})\in \mathrm{cl}\Lambda$, the mapping Σ is continuous at any point of the set $\Omega_{0}:=\{0\}\times \mathrm{cl}\Lambda$. We will show that Σ is inner semi-continuous on $\Omega \setminus \Omega_{0}$. To see this fix an arbitrary $\bar{w}=(\bar{y},\bar{x},\bar{u})\in \Omega \setminus \Omega_{0}$ and then any $\bar{v}\in \Sigma (\bar{y},\bar{x},\bar{u})$. Let $O_{\bar{v}}$ be any open set containing $\bar{v}$.

First, assume that $∥\bar{v}-\bar{u}∥<\ell ∥\bar{y}∥$. As $\bar{v}\in {I\;B}_{\ell ∥\bar{y}∥}(\bar{u})\subset {I\;B}_{a/2}(\bar{u})$ and $\bar{y}\in {I\;B}_{\beta }(0)\subset {I\;B}_{b/2}(0)$ the mapping $G_{\bar{x},\bar{u}}$ is regular at $\bar{v}$ for $\bar{y}$ with the constant *κ* (cf. Corollary 2.5). Thus the mapping $\Phi :=G_{\bar{x},\bar{u}}(\cdot )-\bar{y}$ is regular at $\bar{v}$ for 0 with the same constant. Define the function *g* for each w=(y,x,u)∈Ω and each $v\in R^{m}$ by

$$g(w,v):=f(x,u)+\nabla_{u}f(x,u)(v-u)-y-f(\bar{x},\bar{u})-\nabla_{u}f(\bar{x},\bar{u})(v-\bar{u})+\bar{y}.$$

Let $S(w):=\{v\in R^{m}\;|\;0\in G_{x,u}(v)-y=\Phi (v)+g(w,v)\}$, w=(y,x,u)∈Ω. The continuity of $\nabla_{u}f$ and the implicit form of the Lyusternik-Graves theorem [4, Theorem 5E.5] imply that there are positive constants $\lambda_{\bar{w}}$ and $\delta_{\bar{w}}$ such that

$$S(w^{\prime })\cap {I\;B}_{\delta_{\bar{w}}}(\bar{v})\subset S(w)+\lambda_{\bar{w}}∥w-w^{\prime }∥{I\;B}_{R^{m}}\;\text{whenever }w,w^{\prime }\in {I\;B}_{\delta_{\bar{w}}}(\bar{w})\cap \Omega .$$

As $S(\bar{w})=\Phi^{-1}(0)∋\bar{v}$, taking $w^{\prime }:=\bar{w}$ we get a function $s:{I\;B}_{\delta_{\bar{w}}}(\bar{w})\cap \Omega \to R^{m}$ such that

$$\begin{array}{rl} & y\in G_{x,u}(s(w))\;\text{and}\;∥s(w)-\bar{v}∥\leq \lambda_{\bar{w}}∥w-\bar{w}∥\\ & \;\text{for each }w=(y,x,u)\in {I\;B}_{\delta_{\bar{w}}}(\bar{w})\cap \Omega . \end{array}$$

As $∥\bar{v}-\bar{u}∥<\ell ∥\bar{y}∥$ and the function *s* is continuous at $\bar{w}$ with $s(\bar{w})=\bar{v}$, there is a neighbourhood $O_{\bar{w}}$ of $\bar{w}=(\bar{y},\bar{x},\bar{u})$ with $O_{\bar{w}}\subset {I\;B}_{\delta_{\bar{w}}}(\bar{w})$ such that

$$s(w)\in O_{\bar{v}}\;\text{and}\;∥s(w)-u∥<\ell ∥y∥\;\text{for each}\;w=(y,x,u)\in O_{\bar{w}}\cap \Omega .$$

Consequently, $s(w)\in G_{x,u}^{-1}(y)\cap {I\;B}_{\ell ∥y∥}(u)\cap O_{\bar{v}}=\Sigma (w)\cap O_{\bar{v}}$ for each $w=(y,x,u)\in O_{\bar{w}}\cap \Omega$. So $\Sigma (w)\cap O_{\bar{v}}\neq \emptyset$ for each $w\in O_{\bar{w}}\cap \Omega$.

On the other hand, if $∥\bar{v}-\bar{u}∥=\ell ∥\bar{y}∥$ then find $\hat{v}\in \Sigma (\bar{w})$ with $∥\hat{v}-\bar{u}∥<\ell ∥\bar{y}∥$ (which exists as we have seen right after the definition of Σ). Since the set $\Sigma (\bar{w})$ is convex and contains both $\hat{v}$ and $\bar{v}$, there exists $\tilde{v}\in \Sigma (\bar{w})\cap O_{\bar{v}}$ such that $∥\tilde{v}-\bar{u}∥<\ell ∥\bar{y}∥$. By the previous case, there is a neighbourhood $O_{\bar{w}}$ of $\bar{w}$ such that $\Sigma (w)\cap O_{\bar{v}}\neq \emptyset$ for every $w\in O_{\bar{w}}\cap \Omega$.

In both the cases we showed that Σ is inner semi-continuous at $\left(\bar{w},\bar{v}\right)$. Hence Σ is inner semi-continuous on whole of Ω. Michael selection theorem [4, Theorem 5J.5] yields a continuous mapping *σ* such that

$$\begin{array}{rl} & \sigma (y,x,u)\in G_{x,u}^{-1}(y)\;\text{and}\;∥\sigma (y,x,u)-u∥\leq \ell ∥y∥\\ & \;\text{for each }(y,x,u)\in {I\;B}_{\beta }(0)\times \mathrm{cl}\Lambda . \end{array}$$

Let c∈(0,β/(ν+1)) and $\Omega_{c}:=\{(z,t,p)\in R^{n+1+d}|t\in S,∥z∥\leq c,∥p∥\leq c\}$. Clearly, for each $(z,t,p)\in \Omega_{c}$ we have $p-C(t)z\in {I\;B}_{\beta }(0)$. Define the function

$$\Omega_{c}∋(z,t,p)⟼u(z,t,p):=\sigma (p-C(t)z,\bar{x}(t),\bar{u}(t)).$$

Then for any t∈S (hence for a.e. t∈[0,T]), the function (z,p)⟼u(z,t,p) is continuous. For every $\left\{(z,p)|(z,t,p)\in \Omega_{c}\text{ for some }t\in S\right\}$, the function S∋t⟼u(z,t,p) is measurable as a composition of a continuous function and a measurable function; and

$$\begin{array}{rl} & ∥u(z,t,p)-\bar{u}(t)∥=∥u(z,t,p)-u(0,t,0)∥\leq \ell (∥p∥+\nu ∥z∥)\\ & \;\text{whenever }(z,t,p)\in \Omega_{c}. \end{array}$$

Choose Δ>0 such that

(33) $$\Delta T(1+\ell \nu )e^{\nu (1+\ell \nu )T}<c.$$

Fix arbitrary functions p(⋅)∈P and r(⋅)∈R with $∥p(\cdot ){∥}_{\infty }<\Delta$ and $∥r(\cdot ){∥}_{\infty }<\Delta$. Consider the initial value problem

(34) $$\begin{array}{rl} \dot{z}(t) & =A(t)z(t)+B(t)(u(z\left(t\right),t,p(t))-\bar{u}(t))+r(t)\\ & \;\text{ for a.e. }t\in [0,T],\;z(0)=0. \end{array}$$

The right-hand side of this differential equation is a Carathèodory function, and also the initial condition $z(0)=0\in \mathrm{int}{I\;B}_{c}(0)$. Hence there is a maximal interval [0,τ]⊂[0,T] such that there exists a solution z(⋅)∈X of (34) on [0,τ] with values in ${I\;B}_{c}(0)$, and if τ<T then ∥z(τ)∥=c. Suppose that τ<T. Then for each t∈[0,τ] we have

$$\begin{array}{rl} & ∥z(t)∥\leq \int_{0}^{t}(\nu ∥z(s)∥+\nu \ell (\Delta +\nu ∥z(s)∥)+\Delta )\;ds\\ & \;<\Delta T(1+\ell \nu )+\nu (1+\ell \nu )\int_{0}^{t}∥z(s)∥\;ds. \end{array}$$

Applying the Grönwall lemma and using (33), we get $∥z(t)∥<\Delta T(1+\ell \nu )e^{\nu (1+\ell \nu )T}<c$ for each t∈[0,τ]. In particular, ∥z(τ)∥<c, a contradiction. Hence τ=T and there exists a solution z(⋅) of (34) on the entire interval [0,T] such that $z(t)\in \mathrm{int}{I\;B}_{c}(0)$ for each t∈[0,T]. Let $v(t):=u(z(t),t,p(t))-\bar{u}(t)$, t∈[0,T]. Then (z(⋅),v(⋅))∈V, z(0)=0, and

$$\begin{array}{l} \dot{z}(t)=A(t)z(t)+B(t)v(t)+r(t),\\ p(t)\in \bar{f}(t)+C(t)z(t)+D(t)v(t)-U_{ad}, \end{array}\;\text{for a.e. }t\in [0,T].$$

Hence (r(⋅),p(⋅))∈H(z(⋅),v(⋅))). As H has a closed convex graph, Robinson-Ursescu theorem implies (ii).

It seems that one can formulate a similar statement when a constant mapping $F\equiv -U_{ad}$ is replaced by a general $F:R^{m}\to R^{d}$ with a closed convex graph, which would be a regularity version of [3, Theorem 13]. This is out of the scope of the current work and is a subject for future research.

## Note

1. It is available on: https://www.mathworks.com/matlabcentral/fileexchange/20952-lcp---mcp-solver--newton-based-?requestedDomain=www.mathworks.com
